# Gulf War Illness Is Associated with Host Gut Microbiome Dysbiosis and Is Linked to Altered Species Abundance in Veterans from the BBRAIN Cohort

**DOI:** 10.3390/ijerph21081102

**Published:** 2024-08-21

**Authors:** Ayushi Trivedi, Dipro Bose, Kelly Moffat, Elisabeth Pearson, Dana Walsh, Devra Cohen, Jonathan Skupsky, Linda Chao, Julia Golier, Patricia Janulewicz, Kimberly Sullivan, Maxine Krengel, Ashok Tuteja, Nancy Klimas, Saurabh Chatterjee

**Affiliations:** 1 Environmental Health and Disease Laboratory, Department of Environmental and Occupational Health, Program in Public Health, Susan and Henry Samueli College of Health Sciences, University of California, Irvine, CA 92697, USA; aktrived@uci.edu (A.T.); diprob@uci.edu (D.B.); 2 CosmosID, Germantown, MD 20874, USA; kelly.moffat@cosmosid.com (K.M.); dana@cosmosid.com (D.W.); 3 Miami VA Healthcare System, Miami, FL 33125, USA; dcohen1@nova.edu; 4 Institute for Neuro-Immune Medicine, Nova Southeastern University, Fort Lauderdale, FL 33314, USA; nklimas@nova.edu; 5 VA Research and Development, VA Long Beach Health Care, Long Beach, CA 90822, USA; jonathan.skupsky@va.gov; 6 San Francisco Veterans Affairs Health Care System, San Francisco, CA 94121, USA; 7 Department of Radiology and Biomedical Imaging, University of California, San Francisco, CA 94143, USA; 8 Department of Psychiatry and Behavioral Sciences, University of California, San Francisco, CA 94143, USA; 9 J. Peters VA Medical Center, Bronx, NY 10468, USA; julia.golier@va.gov; 10 Psychiatry Department, Icahn School of Medicine at Mount Sinai, 1428 Madison Ave, New York, NY 10029, USA; 11 Department of Environmental Health, Boston University School of Public Health, 715 Albany St. T4W, Boston, MA 02130, USA; paj@bu.edu (P.J.);; 12 Department of Neurology, Boston University Chobanian and Avedisian School of Medicine, Boston, MA 02130, USA; mhk@bu.edu; 13 Division of Gastroenterology, School of Medicine, University of Utah, Salt Lake City, UT 84132, USA; ashok.tuteja@hsc.utah.edu; 14 Geriatric Research and Education Clinical Center, Miami VA Heathcare System, Miami, FL 33125, USA; 15 Department of Medicine, Infectious Disease, UCI School of Medicine, Irvine, CA 92697, USA

**Keywords:** Gulf War Illness, veteran, bacteriome, chronic fatigue, bacterial metabolism, Multidimensional Fatigue Inventory, *Lachnospiraceae*, *Coprococcus*, *Eisenbergiella*

## Abstract

Gulf War Illness (GWI) is a debilitating condition marked by chronic fatigue, cognitive problems, pain, and gastrointestinal (GI) complaints in veterans who were deployed to the 1990–1991 Gulf War. Fatigue, GI complaints, and other chronic symptoms continue to persist more than 30 years post-deployment. Several potential mechanisms for the persistent illness have been identified and our prior pilot study linked an altered gut microbiome with the disorder. This study further validates and builds on our prior preliminary findings of host gut microbiome dysbiosis in veterans with GWI. Using stool samples and Multidimensional Fatigue Inventory (MFI) data from 89 GW veteran participants (63 GWI cases and 26 controls) from the Boston biorepository, recruitment, and integrative network (BBRAIN) for Gulf War Illness, we found that the host gut bacterial signature of veterans with GWI showed significantly different Bray–Curtis beta diversity than control veterans. Specifically, a higher *Firmicutes* to *Bacteroidetes* ratio, decrease in *Akkermansia* sp., *Bacteroides thetaiotamicron*, *Bacteroides fragilis*, and *Lachnospiraceae* genera and increase in *Blautia*, *Streptococcus*, *Klebsiella*, and *Clostridium* genera, that are associated with gut, immune, and brain health, were shown. Further, using MaAsLin and Boruta algorithms, *Coprococcus* and *Eisenbergiella* were identified as important predictors of GWI with an area under the curve ROC predictive value of 74.8%. Higher self-reported MFI scores in veterans with GWI were also significantly associated with an altered gut bacterial diversity and species abundance of *Lachnospiraceae* and *Blautia*. These results suggest potential therapeutic targets for veterans with GWI that target the gut microbiome and specific symptoms of the illness.

## 1. Introduction

Gulf War Illness (GWI), a chronic multi-symptom illness, has affected and altered the quality of life for thousands of US Gulf War veterans (GWV) [1]. Approximately 700,000 troops from the US were deployed to the Persian Gulf, and the estimates are that this illness afflicts a third of those who were deployed [2]. Shortly following the end of the Gulf War in 1991, veterans began reporting a plethora of health symptoms from multiple organ systems including the central nervous system and the gastrointestinal system [2]. Over the years, significant research has accumulated that supports a link between deployment to the Persian Gulf during Operation Desert Shield/Operation Desert Storm, environmental exposures from the war, and the development of GWI-related symptoms [2,3]. More specifically, researchers have identified a link between specific toxicant exposures, including pesticides, anti-nerve gas pills (pyridostigmine bromide—PB), and nerve agent chemical weapons (sarin/cyclosarin), during deployment and the development of GWI-related health symptoms [2,4].

Since our first published report of a pronounced role of gut microbiome (bacteria and viruses) alterations in cell and animal models of GWI and related disease pathology in 2017 [5] (bacteriome) and 2019 (virome), our lab has advanced the understanding of a microbiome role in GWI and, accordingly, conducted research for the development of targeted therapeutics that target the gut–brain axis rather than only the GI (gastrointestinal) functions owing to the complex plethora of complications in GWI [6]. Our series of publications provide strong evidence of such disease etiology attributable to bacteriome diversity [5], butyrate’s role in gut dysbiosis [7], microbiome-associated enteric glial cell activation and neuroinflammation [8], the role of the gut virome in GWI [9], gut microbiome alterations in GWI veterans, and [10] microbiome alterations in GWI symptom persistence [11] and TLR-antagonism in GWI pathology and cure [12]. There is strong clinical evidence that alterations in the gut microbiome lead to GI disturbances, chronic fatigue syndrome, metabolic complications (obesity, type-2 diabetes, insulin resistance, and nonalcoholic fatty liver disease—NAFLD), and even neuro-cognitive disturbances (gut–brain axis) [13,14]. GWI-associated changes in bacteriome and virome signatures were strongly associated causally with gastrointestinal (GI) inflammation, the release of damage-associated molecular patterns (DAMPs) such as HMGB1, and proinflammatory cytokines. Importantly, we found that serum levels of IL6, which are increased in veterans with GWI, were associated with bacteriome and virome change in the mouse model following exposure to the GW chemicals pyridostigmine bromide (PB) and permethrin [8,9].

Gastrointestinal (GI) symptoms are hallmarks of GWI [15,16]. In an earlier preliminary pilot study from our team [10], we assessed the relationships between GWI, GI symptoms, gut microbiome, and inflammatory markers in GWI among veterans from the Boston Gulf War Illness Consortium (GWIC) cohort. Specifically, Gulf War (GW) controls who did not meet the criteria for GWI, had a greater abundance of *Firmicutes*. In comparison, the GWI + GI group had a greater abundance of the phyla *Bacteroidetes*, *Actinobacteria*, *Euryarchaeota*, and *Proteobacteria* as well as higher abundances of the families *Bacteroidaceae*, and *Erysipelotrichaceae*, all of which are associated with systemic inflammatory response. The virome consists of a diverse collection of viruses that infect our own cells as well as other commensal organisms, directly impacting our well-being [17]. Interestingly, we have recently shown that the alteration of a healthy virome occurs in a GWI mouse model [9]. Further, there is evidence that GW chemical exposure caused an alteration to the gut bacteriome and virome that was related to GI inflammation, increased IL6 release, a significant decrease in blood–brain barrier (BBB) tight junction protein Claudin-5, and neurotoxicity. Earlier, we conducted a pilot study that focused on the gut microbiome in veterans and its association with GWI in a small cohort that could only analyze family-level abundance, a relatively simplistic assessment, and extending those findings in a more comprehensive analysis in a larger GW veteran cohort was necessary [10]. Additionally, translating the body of preclinical research to the veterans with GWI cohort remains the focus among GWI researchers, and the present report further advances our understanding of the role of the host gut microbiome in GWI etiology as we conducted a detailed microbiome analysis using stool samples from a large biorepository cohort called BBRAIN for GWI representing eighty-nine veterans [18]. The study will also help to identify targeted probiotics, bacterial metabolites, or small molecule intermediates for therapy in future clinical trials as new results identify several bacterial species that are differentially altered in veterans with GWI.

## 2. Materials and Methods

Prospective biospecimen data collection [18]: Briefly, study eligibility included serving in the 1990–1991 Gulf War without any medical exclusions that could otherwise result in case criteria required for participation. Useable stool samples and corresponding Multidimensional Fatigue Inventory (MFI) scores were obtained from 89 participants for the present study, which included 63 GWI cases (referred to as GWI group) and 26 deployed controls without GWI (referred to as the control group).

GWI case status was defined by the Kansas health symptom questionnaire [19]. The Kansas case definition requires GWI cases to endorse multiple mild or one or more moderate-to-severe chronic symptoms in at least three of six statistically defined symptom domains: fatigue/sleep problems, somatic pain, neurological cognitive, mood symptoms, gastrointestinal symptoms, respiratory symptoms, and skin abnormalities. Veterans were excluded from being considered GWI cases, for purposes of the research study, if they reported being diagnosed by a physician with medical or psychiatric conditions that would account for their symptoms or interfere with their ability to report their symptoms.

Specific exclusion criteria included such medical conditions as uncontrolled diabetes, heart disease other than hypertension, stroke, lupus, multiple sclerosis, cancer, liver disease, chronic infection, or serious brain injury. Veterans were also excluded if they reported being diagnosed with schizophrenia or bipolar disorder or if they had been hospitalized in the past 2 years for alcohol/drug dependence, depression, or post-traumatic stress disorder (PTSD). Participants with a current or past history of PTSD or depression in the past 2 years were not excluded from participation if they had not been hospitalized for these conditions.

Inclusion and exclusion criteria: Veterans were included if they met the criteria for one of the two participant groups (case or control) using the Kansas questionnaire and did not meet any exclusion criteria.

Stool sample procedures: Stool sample analysis is currently the only biological sample where information about the gut microbiome can be analyzed. Each participant was given specific written and oral instructions on how to systematically collect their stool samples according to DNA Genotek Omnigene GUT stool kit (Ottawa, Ontario, Canada) instructions. Pre-assembled stool collection kits were provided to the participants, and using the collection kit, participants collected stool at home and shipped it directly to the Boston University Medical Campus General Clinical Research Unit (GCRU) for storage. The collection tubes included a stabilizing solution that kept the samples stable at room temperature during transit. Samples were stable for 15 days at room temperature and very well preserved over an extended period of time at −4 °C.

Quality of life and fatigue scores: Clinical assessments for the current analysis included the Kansas Gulf War and Health Questionnaire and the Multidimensional Fatigue Inventory (MFI-20) questionnaire [20].

### 2.1. IRB Approval and Informed Consent

For the original BBRAIN study, all participants signed informed consent to share data for future studies (IRB # H-37828) and a separate IRB protocol was approved for this study (BU IRB # H-42872). The microbiome analysis was performed using the VA Long Beach IRB # 1738453-2 which was determined not to be human subjects research.

#### 2.1.1. Gut Microbiome Analysis

##### DNA Extraction and Quantification

DNA from samples was isolated using the QIAGEN DNeasy PowerSoil Pro Kit (Qiagen, Hilden, Germany), according to the manufacturer’s protocol. DNA samples were quantified using Qubit 4 fluorometer and Qubit™ dsDNA HS Assay Kit (Thermofisher Scientific, Waltham, MA, USA).

##### Library Preparation and Sequencing Methods

For 16S amplicon sequencing, 5 ng of isolated genomic DNA was sampled. Libraries are constructed by amplification via PCR with primers. Sequencing was performed on Illumina Miseq platform (Illumina Inc., San Diego, CA, USA) 2 × 250 bp.

##### Bioinformatics Analysis Methods

The CosmosID-HUB Microbiome’s 16S workflow implements the DADA2 algorithm [21] as its core engine and utilizes the Nextflow ampliseq pipeline (v19.10.0.5170) definitions to run it on the CosmosID-HUB cloud infrastructure [22,23]. Briefly, primer removal is done with Cutadapt, and quality trimming parameters were passed to DADA2 to ensure that the median quality score over the length of the read exceeds a certain Phred score threshold. Within DADA2, forward and reverse reads were each trimmed to a uniform length based on the quality of reads in the sample—higher quality data will generally result in longer reads. DADA2 used machine learning with a parametric error model to learn the error rates for the forward and reverse reads, based on the premise that correct sequences should be more common than any particular error-variant. DADA2 then applied its core sample inference algorithm to the filtered and trimmed data, applying these learned error models. Paired-end reads were merged if they had at least twelve bases of overlap and were identical across the entire overlap. The resulting table of sequences and observed frequencies was filtered to remove chimeric sequences (those that exactly match a combination of more prevalent “parent” sequences). Taxonomy and species-level identification (where possible) were conducted with DADA2′s naive Bayesian classifier, using the Silva version 138 database. Lastly, the predicted functional potential of the community was profiled using PICRUSt2 [23,24]. Briefly, the PICRUSt2 (Phylogenetic Investigation of Communities by Reconstruction of Unobserved States) is a tool that predicts functional capabilities and abundances of a microbial community based on the observed amplicon (marker gene) content [23]. Functional capabilities are given by Enzyme Commission (EC) classifiers or MetaCyc ontologies, and these can be aggregated to predict pathways that are likely present in a given sample [25].

##### Figure Generation and Statistical Analysis

Relative abundance stacked bars: Stacked bar figures were generated using phylum-, genus-, species-, and strain-level filtered matrices for bacteria from CosmosID-HUB. Stacked bar figures for each group were generated using the R packages ggplot2 and ggpubr [26].

Heatmaps: Heatmaps were creating using the pheatmap R package generated using the phylum, genus, species, and strain matrices for bacteria from CosmosID-HUB. Hierarchical clustering and dendrograms are generated using the hclust and dist functions from the R stats package, with default parameters using Euclidean distance and complete linkage.

Alpha diversity boxplots (with Wilcoxon rank-sum test): Alpha diversity boxplots were calculated from the phylum-, genus-, species-, and strain-level abundance score matrices from CosmosID-HUB analysis. Chao1, Simpson, and Shannon alpha diversity metrics were calculated in R using the R package Vegan. Wilcoxon rank-sum tests were performed between groups using the R package ggsignif. Boxplots with overlaid significance in *p*-value format were generated using the R package ggpubr.

Beta diversity Principal coordinate analysis (PCoA) (with PERMANOVA): Beta diversity PCoA were calculated from phylum-, genus-, species-, and strain-level matrices for bacteria from CosmosID-HUB. Bray–Curtis dissimilarity was calculated in R using the vegan package with the function vegdist, and PCoA tables were generated using ape’s function PCoA [27]. PERMANOVA tests for each distance matrix were generated using vegan’s function adonis, and beta dispersion was calculated and compared using the ANOVA method for the betadisper function from vegan. Plots were visualized using the R package ggpubr.

DESeq2: DESeq2 used a negative binomial distribution model to estimate differential abundance between cohorts based on count data [28]. The algorithm assumes that most features in microbiome data should not vary greatly between conditions, so it preferentially highlights features that (a) are highly expressed/prevalent, and (b) have large fold changes in prevalence and are statistically significantly different [21]. The figures presented are annotated with some of the most significant features.

MaAsLin: MaAsLin was implemented using the R package MaAsLin2. MaAsLin (Microbiome Multivariable Associations with Linear Models) is designed to assess multivariable association with microbiome community features with complex metadata [29]. MaAsLin performs generalized linear and mixed models to accommodate a wide range of studies and data types (counts or relative abundance), including longitudinal and cross-sectional study designs. It was used to identify significant associations of metadata of interest with individual taxa.

Boruta: Boruta is a wrapper around a random forest machine learning algorithm. Boruta improves the baseline algorithm by calculating shadow variables from the data itself in order to determine whether variables are important in classifying a binary response. Boruta performs a top-down search for relevant features by comparing the importance of the original attributes to that of random permutations of the attributes. Irrelevant features were progressively eliminated to stabilize the test set.

ROC curves: Receiver operator characteristic (ROC) curves use test sensitivity and specificity to determine if a metric improves the classification of a binary response variable [30]. The R package pROC was used to plot the ROC curve.

For both Boruta and ROC curves, data were split into test and train sets to optimize parameters using the training set before running the algorithm on the test set.

Statistical significance for Chao1 and Shannon α-diversity between the control and GWI group was calculated using the R package (version 4.3.2). A Welch two-sample *t*-test was performed to calculate the statistical difference for species-level abundance and total MFI-20 scores and all subscales using GraphPad Prism (version 10.2.2 (397)), (San Diego, CA, USA). Pearson correlation between α-diversity, selected bacterial species, and the fatigue score was determined with 95% confidence bands using GraphPad Prism software. All data are represented as mean + standard deviation and for all analyses, *p*  <  0.05 was considered statistically significant. Individual *p*-values have been assigned to each figure and dataset for precise interpretation.

Other statistical analyses: Chi-square, Fisher’s exact test, and Wilcoxon rank-sum tests were used to compare demographic variables (age, sex, race, and education) between GWI cases and controls. Welch two-sample t-test was performed to calculate the statistical difference for species-level abundance and total MFI-20 scores and all subscales using GraphPad Prism (version 10.2.2 (397)), (San Diego, CA, USA). Pearson correlation between α-diversity, selected bacterial species and fatigue scores (General, Physical, Average, and Total) was determined with 95% confidence bands using GraphPad Prism software. All data are represented as mean ± standard deviation, and *p*  <  0.05 was considered statistically significant for all analyses. For microbiome and correlation analyses, individual *p*-values have been assigned to each figure and dataset for precise interpretation.

## 3. Results

Study Cohort: Samples from this study were used from the larger BBRAIN repository and participants were on average 57 years old, 71% male, 70% White, and had 15 years of education. The GWI cases (*n* = 63) and controls (*n* = 26) did not significantly differ by age, sex, race, or education level (Table 1).

Microbiome abundance and unique niches within group (alpha diversity) and between groups (Beta diversity) were altered in veterans with GWI when compared to the control group.

**Figure 1:**
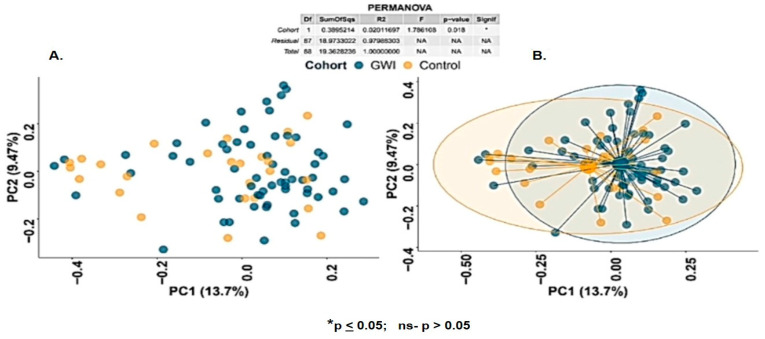
(**A**,**B**) β-diversity analysis (Bray–Curtis) of altered gut bacteriome at species level in the control group (deployed GW Veterans with no GWI) and the GWI group (deployed GW veterans with GWI symptoms). *p* < 0.05 was considered as statistically significant.

The host gut microbiome consists of trillions of bacteria, viruses, and fungi that together constitute a unique homeostatic microenvironment. The host’s microbiome diversity, richness, and abundance play a crucial role in the individual’s overall well-being. Alpha diversity metrics summarize the structure of an ecological community, herein the control sample or the GWI sample, respectively, with respect to its richness (the number of taxonomic groups), evenness (the distribution of the abundance of the groups), or both [31]. Since many perturbations to a community affect the alpha diversity of a community, summarizing and comparing community structure via alpha diversity is a ubiquitous approach to analyzing bacterial communities within a particular group. The evenness and richness of bacterial species and communities within GWI and control groups as measured by Shannon diversity indices were markedly similar. However, the richness spread was lower in the GWI group compared to the control group (statistically not significant, *p* = 0.44) (Supplementary Figure S1). Beta-diversity analysis is the analysis of the microbial composition in each group/pair; in the present report these are the control and the GWI microbiome pairs [32]. The basis of beta-diversity analysis is that one can link the overall taxonomic or functional diversity pattern to the unique bacterial niche in one particular group when compared to the pair group (control). An accurate and reliable similarity or distance metric among microbiomes is the basis for deducing the microbial beta-diversity. Statistical or geometric approaches like Bray–Curtis, Jaccard, and Jensen–Shannon divergence calculate such distances mainly by counting the overlapped components [32]. Bray–Curtis species beta diversity was significantly different between the GWI and control groups (Figure 1) (*p* = 0.018). The GWI samples exhibit closer clustering, whereas the controls display greater dispersion, suggesting a community-level alteration in GWI that fosters a higher similarity in the stool microbiome of the GWI veterans significantly different in profile than the control microbiome. The above significant change in beta diversity was also observed at the family and genus level (Supplementary Figures S2 and S3).

### 3.1. GWI Resulted in an Altered Firmicutes–Bacteroidetes Ratio and Expression of Unique Family and Genus Abundance

**Figure 2:**
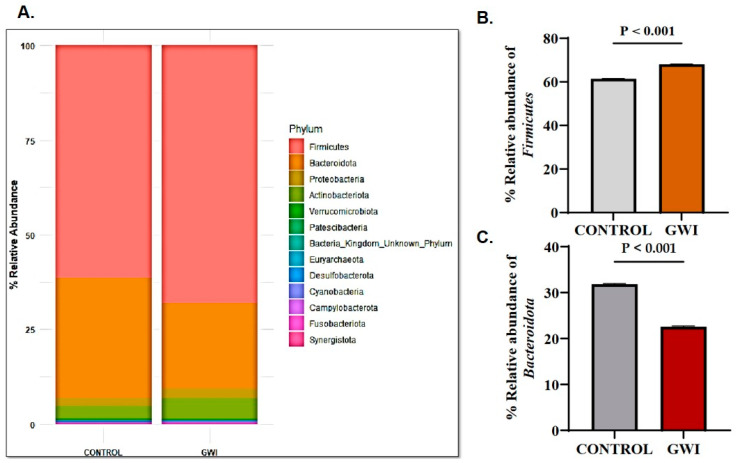
(**A**) Stacked bar representation of altered gut bacteriome at phylum level in the control group (deployed GW veterans with no GWI) and the GWI group (deployed GW veterans with GWI symptoms). (**B**) Bar graph representation of relative abundances of phylum *Firmicutes* in the control (grey bar) and GWI (brown bar) groups. (**C**) Bar graph representation of relative abundances of phylum *Bacteroidota* in the control (grey bar) and GWI (dark red bar) groups. *p* < 0.05 was considered as statistically significant.

Though the relative abundance of respective phyla, their richness, and evenness have been shown in earlier microbiome studies, they may not be significant players in downstream species-induced modulations of host function. As innovative technologies for assessing microbiome functionality evolve, the role of the *Bacteriodetes*–*Firmicutes* ratio has assumed a prominent role and is associated with several disease conditions [33]. A differential abundance in *Firmicutes* over *Bacteroidetes* is associated with obesity, metabolism-associated steatosis liver disease and the progression of IBS/IBD (Irritable Bowel Syndrome/Inflammatory Bowel Disease) [33,34]. Further, an increased abundance has been shown to be associated with fatigue in an exercise-induced swim test in mice [35]. In this study, there was a marked increase in *Firmicutes* abundance in the GWI group when compared to the control group (Figure 2A,B) (*p* < 0.001), while there was a significant decrease in *Bacteroidetes* abundance in the GWI group (Figure 2C) (*p* < 0.001). A heat map showing the distribution of various phyla in the cohort also showed the same trend with a significantly higher distribution of *Firmicutes* in the GWI group when compared to controls (Supplementary Figure S4). Interestingly, another prominent phylum, *Verrucomicrobiota*, did not show any difference in abundance (Supplementary Figure S4). A stacked bar analysis of genus abundance showed differential expression amongst *Blautia*, *Prevotella*, *Ruminococcus*, *Bifidobacterium*, *Alistipes*, *Holdemanella*, and *CAG_352*, from the family *Ruminococcaceae* (Supplementary Figure S5). To further elucidate the genus level analysis of the differential abundance, a DESeq2 analysis was performed. The package DESeq2 provides methods to test for differential expression using negative binomial generalized linear models; the estimates of dispersion and logarithmic fold changes incorporate data-driven prior distributions. This DESeq2 plot exhibited enriched taxa with log2 fold-change plotted against log2 abundance. Highlighted points indicate features where the adjusted *p*-value is significant. The top group (red) shows features enriched (or up) in GWI as compared to the control. The bottom group (blue) shows features depleted (or down) in GWI as compared to the control. As shown in Supplementary Figure S6, twenty-nine specific genera were significantly enriched in the GWI group, whereas nine specific genera were significantly decreased in the GWI group when compared to the control. Notably, more than 169 genera did not show significant alteration (Supplementary Figure S6).

### 3.2. GWI Cases Expressed a Differential Abundance of Host Species While Presenting a Unique Species Signature Compared to Non-GWI Controls

**Figure 3:**
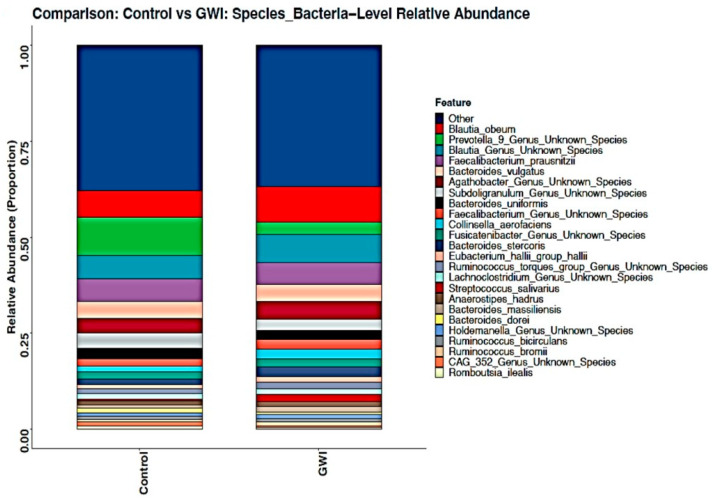
Stacked bar representation of altered gut bacteriome at species level in the control group (deployed GW veterans with no GWI) and the GWI group (deployed GW veterans with GWI symptoms). *p* < 0.05 was considered as statistically significant.

**Figure 4:**
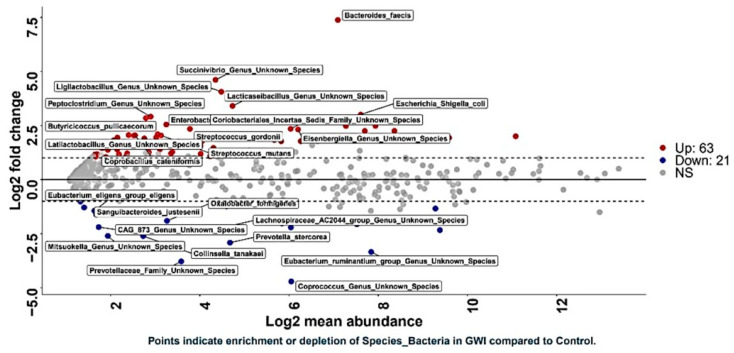
DESeq2 plot shows enriched taxa with log2 fold change plotted against log2 abundance at species level in control groups (deployed GW veterans with no GWI) and GWI group (deployed GW veterans with GWI symptoms). The red points show features significantly enriched in the GWI group and blue points show features significantly enriched in the control group. *p* < 0.05 was considered as statistically significant.

**Figure 5:**
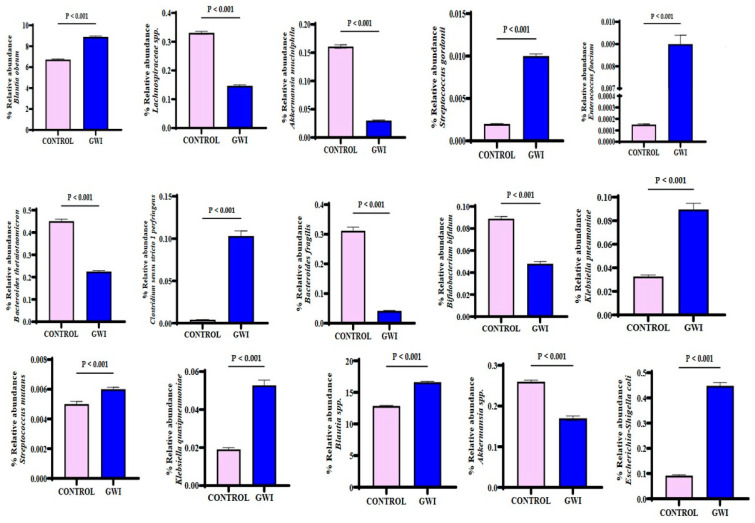
Bar graph representation of relative abundances of gut bacteriome at the genus level in the control group (deployed GW veterans with no GWI) and the GWI group (deployed GW veterans with GWI symptoms). *p* < 0.05 was considered as statistically significant.

Host microbial species play a significant role in modulating the downstream functions of the microbiome. Studies in mice models for GWI have consistently shown the role of species abundance, differential expression, and their released metabolites in disease etiology [6]. A stacked bar analysis showed that *Blautia obeum*, *Prevotella* sp., *Agathabacter* sp., *Subdoligranulum* sp., *Bacteriodes uniformis*, *Streptococcus salivarius*, *Bacteroides dorei*, *Holdemanella* sp., and *CAG 352* sp. were differentially abundant in the samples analyzed (Figure 3). A DESeq2 analysis showed that sixty-three unique bacterial species were significantly increased in abundance in the GWI group. In contrast, twenty-one unique species were significantly decreased in the same group when compared to controls (Figure 4). A deep-down analysis of linear scale fold change (percentage abundance) showed that nine unique species (*Blautia obeum*, *Streptococcus gordonii*, *Enterococcus faecium*, *Clostridium perfringens*, *Klebsiella pneumoniae*, *Streptococcus mutans*, *Klebsiella quasipneumoneae*, *Blautia* spp., and *Escherichia*-*Shigella coli*) were significantly increased in abundance in the GWI group when compared to controls (Figure 5) (*p* < 0.001). Interestingly, several bacteria with probiotic roles (*Lachnospiraceae* spp., *Bacteriodetes thetaiotamicron*, *Bacteriodes fragilis*, *Bifidobacterium bifidum*, and *Akkermansia* spp.) were significantly decreased in the GWI group when compared to the control group (Figure 5) (*p* < 0.001).

**Figure 6:**
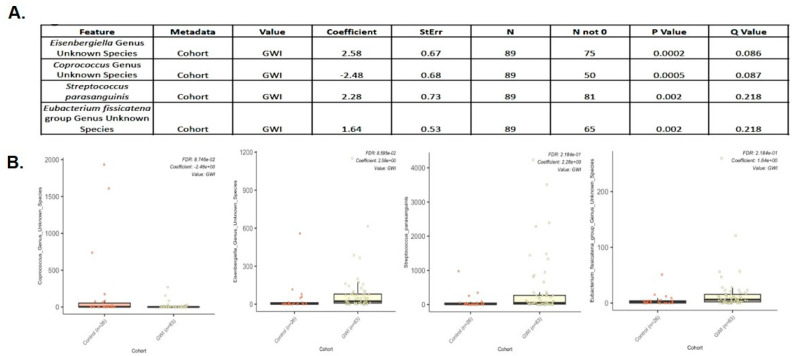
(**A**) Tabular representation of four bacterial species identified by the MaAsLin analysis as significantly different for the GWI group (deployed GW veterans with GWI symptoms) compared to the control group (deployed GW veterans with no GWI) according to default parameters (*p* < 0.05 and Q < 0.25). The column ‘Metadata’ indicates the variable tested and ‘Value’ indicates for which value of the variable the specific species is significantly enriched. A positive coefficient indicates a positive association with the control group and a negative coefficient indicates a positive association with the GWI group. (**B**) Box plot showing the relative abundances of four bacterial species identified by the MaAsLin analysis. In the boxplot, ‘Value’ indicates for which variable the false discovery rate (FDR) and coefficient values are being reported. *p* < 0.05 was considered as statistically significant.

**Figure 7:**
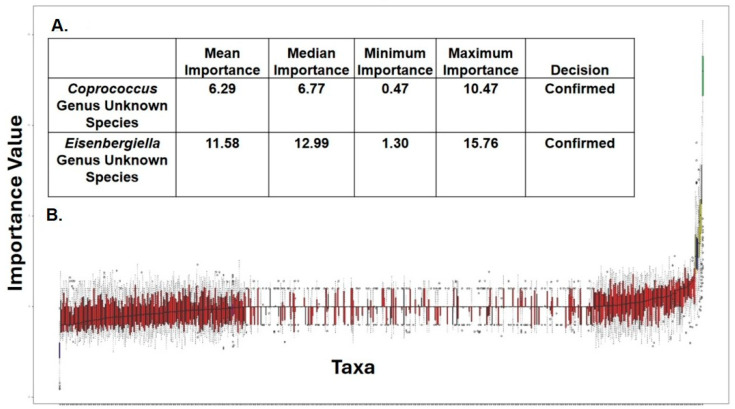
(**A**) Table and plot showing all bacterial species ranked by order of importance according to the Boruta algorithm. (**B**) The importance of the bacterial species is depicted as importance values. Of all species identified, two were important (labeled in green in the plot) in the GWI group (deployed GW veterans with GWI symptoms) compared to the control group (deployed GW veterans with no GWI). The features in yellow are tentative and those in red are rejected as important.

**Figure 8:**
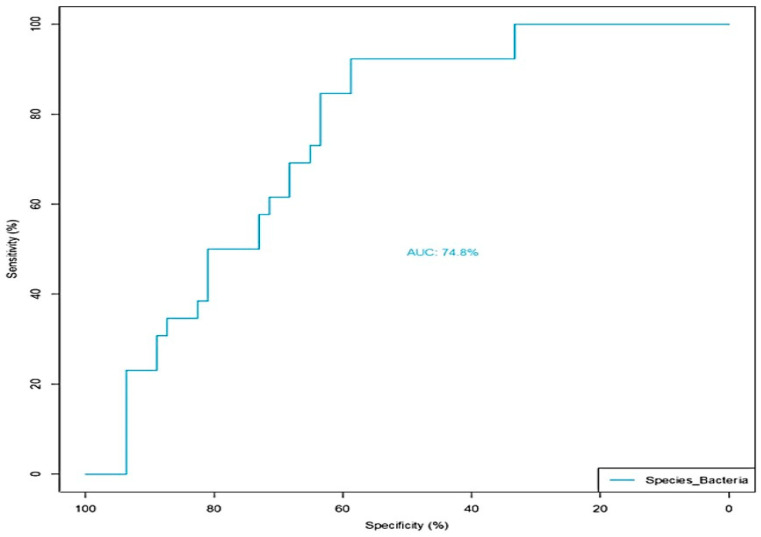
Receiver operator characteristic (ROC) curve analysis depicting the two important species *Coprococcus* and *Eisenbergiella* as an important predictor of the Gulf War Illness condition as identified from the Boruta analysis in the control group (deployed GW veterans with no GWI) and the GWI group (deployed GW veterans with GWI symptoms). *p* < 0.05 was considered as statistically significant.

MaAsLin2, a next generation of MaAsLin (Microbiome Multivariable Association with Linear Models) is a comprehensive R package for efficiently determining multivariable association between clinical metadata and microbial meta-omics features [29]. MaAsLin2 relies on general linear models to accommodate most modern epidemiological study designs, including cross-sectional and longitudinal, along with various filtering, normalization, and transformation methods [29]. We used MaAsLin to add more rigor in analyzing the species diversity in our cohort. MaAsLin identified four taxa as significantly different for the treatment group according to default parameters (*p* < 0.05 and Q < 0.25) (Figure 6). The column ‘Metadata’ indicates the variable tested and ’Value’ indicates for which value of the variable the specific species is significantly enriched. A positive coefficient indicates a positive association with the GWI group, and a negative coefficient indicates a positive association with the control group. In addition, *Coprococcus* sp. was significantly decreased in abundance in the GWI cohort while new species *Eisenbergialla* spp., *Streptococcus parasanguinis*, and *Eubacterium fissicatena* were decreased (Figure 6A,B). To further analyze the bacterial species ranked by order of importance, we used the Boruta algorithm. The Boruta algorithm was developed to identify all relevant variables within a classification framework. In each run of this algorithm, the set of predictor variables is doubled by adding a copy of each variable. The values of those shadow variables are generated by permuting the original values across observations, maintaining their original distribution but destroying their importance in relationship with the outcome, in this case the disease (GWI) [36]. This allows Boruta to compare the importance of the shadow variables to each true feature from the data, and if the true feature is higher, it will be retained as important to differentiating between GWI and control. The Boruta approach has been used in >100 studies, including omics datasets resulting from gene expression and microbiome data analysis [37,38]. Of all species identified, *Coprococcus* and *Eisenbergiella* were most important in identifying GWI samples (labeled in green in the Boruta plot in Figure 7). Features in yellow are tentative and those in red are rejected as important. The table above the plot shows the two features identified as very relevant and gives their importance values, suggesting that *Coprococcus* and *Eisenbergiella* abundance in the cohort may be a true predictor of GWI (Figure 7A,B). Further, these two important predictor taxa identified by Boruta improve the area under the curve (AUC) to 74.8%. For this receiver operating characteristic (ROC) curve, only taxa identified as important by Boruta were included in the model (the unidentified *Coprococcus* and *Eisenbergiella* species). Using these two taxa in the model improved the AUC to 74.8% compared to using the entire detected microbial community. This suggests that these two taxa may be biomarkers of GWI as compared to control samples (Figure 8).

### 3.3. GWI Cases Presented a Unique Bacterial Enzyme Expression Profile with a Differential Abundance of Peptidylprolyl Isomerase and NADH Ubiquinone Reductase, a Mitochondrial Respiration Enzyme

**Figure 9:**
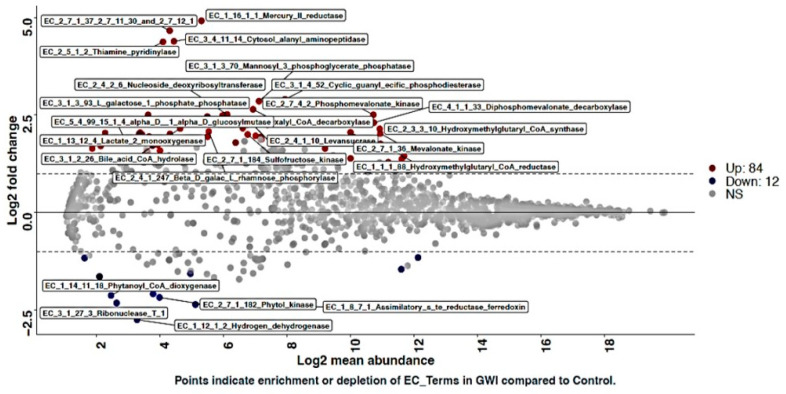
DESeq2 plot shows enriched taxa with log2 fold change plotted against log2 abundance of altered Enzyme Commission enzymes in the control group (deployed GW veterans with no GWI) and the GWI group (deployed GW veterans with GWI symptoms). The red points show features significantly enriched in the GWI group and blue points show features significantly enriched in the control group. *p* < 0.05 was considered as statistically significant.

Using DESeq2 analysis, we studied the unique gut bacterial enzymes that may have been associated with the disease etiology. Eighty-four unique enzymes were upregulated in the GWI cases while twelve enzymes were significantly downregulated when compared to the controls (Figure 9). Out of the eighty-four enzymes, Peptidylprolyl isomerase and NADH Ubiquinone Reductase decreases in the GWI cases were further confirmed using a heat map analysis (Supplementary Figure S7).

### 3.4. GWI Cases Presented a Unique Bacterial Biochemical Pathway That Was Influenced by Microbiome Profile

**Figure 10:**
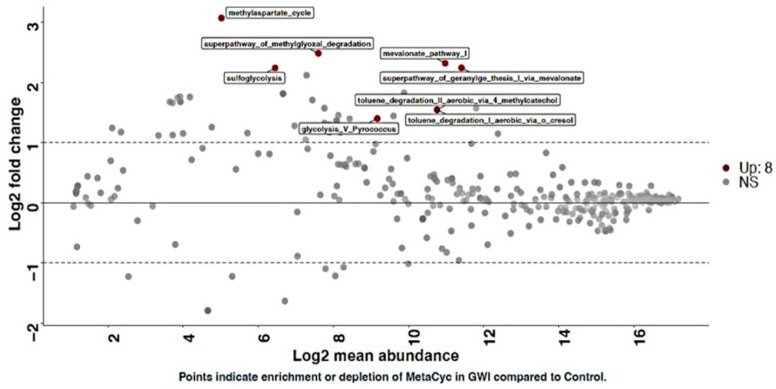
DESeq2 plot shows enriched taxa with log2 fold change plotted against log2 abundance of altered MetaCyc metabolic pathways in the control group (deployed GW veterans with no GWI) and the GWI group (deployed GW veterans with GWI symptoms). The red points show features significantly enriched in the GWI group and blue points show features significantly enriched in the control group. *p* < 0.05 was considered as statistically significant.

MetaCyc is a curated database of experimentally elucidated metabolic pathways from all domains of life. MetaCyc contains pathways involved in both primary and secondary metabolism, as well as associated metabolites, reactions, enzymes, and genes. The goal of MetaCyc is to catalog the universe of metabolism by storing a representative sample of each experimentally elucidated pathway; in this case, we focused on the host bacterial metabolic pathways that may influence the disease etiology in GWI [39]. Results showed that eight unique biochemical pathways related to host bacterial metabolism were significantly enriched in the GWI cohort, including Glycolysis V (*Pyrococcus*), the Methylaspartate cycle, Toluene degradation, and the mevalonate pathway. Though these pathways are identified, the exact roles of these perturbations remain unknown (Figure 10).

### 3.5. GWI Cases Altered Microbiome Diversity and Species Abundance Correlated with MFI Fatigue Scores

**Figure 11:**
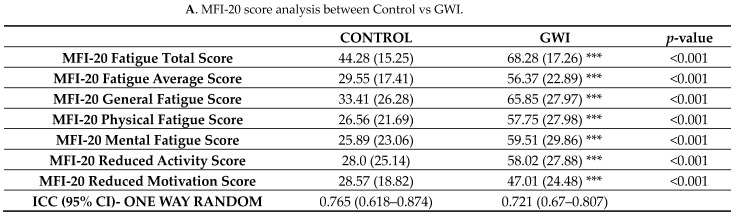

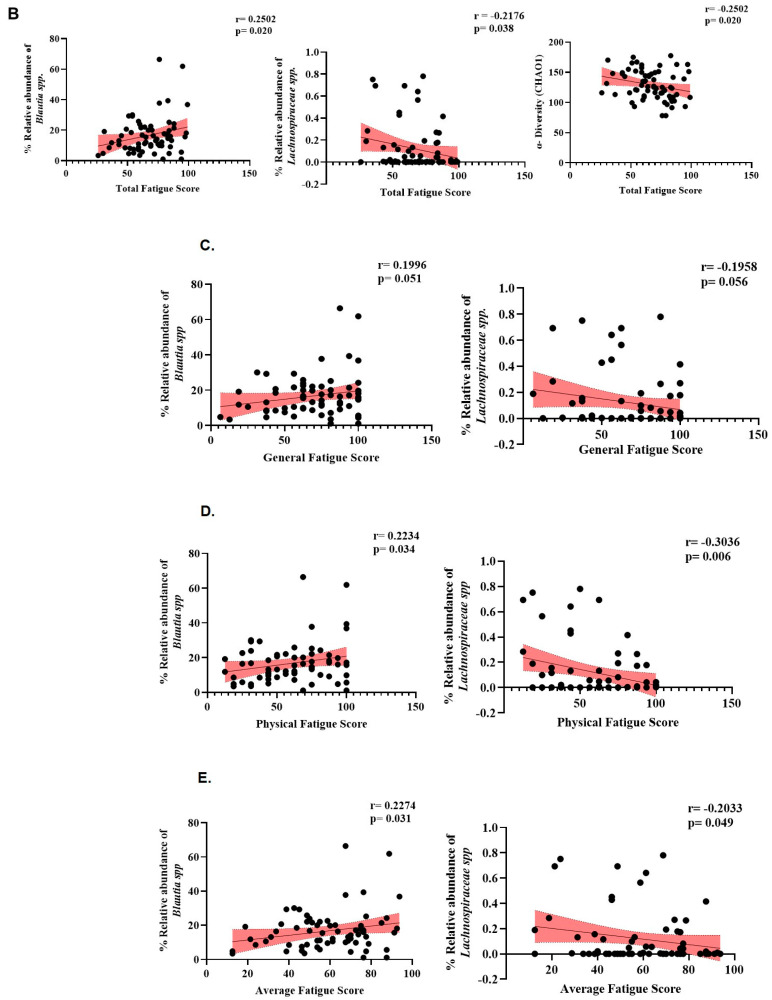
((**A**)—table) Tabular representation of Multidimensional Fatigue Inventory-20 (MFI-20) fatigue scores in the control group (deployed GW veterans with no GWI) and GWI group (deployed GW veterans with GWI symptoms). Data are represented as mean + SD. Statistical difference was calculated using Student’s *t*-test with Welch’s correction between the control and GWI groups. *p* < 0.05 was considered statistically significant and denoted by *. (**B**) Pearson correlation plot between % relative abundance of species *Blautia* and *Lachnospiraceae*, and α-diversity (Chao1) with Total Fatigue Score in the GWI group (deployed GW veterans with GWI symptoms). (**C**) Pearson correlation plot between % relative abundance of species *Blautia* and *Lachnospiraceae* with General Fatigue Score in GWI group (deployed GW veterans with GWI symptoms). (**D**) Pearson correlation plot between % relative abundance of species *Blautia* and *Lachnospiraceae* with Physical Fatigue Score in the GWI group (deployed GW veterans with GWI symptoms). (**E**) Pearson correlation plot between % relative abundance of species *Blautia* and *Lachnospiraceae* with Average Fatigue Score in GWI group (deployed GW veterans with GWI symptoms). *** *p* < 0.001; * *p* < 0.05.

Having elucidated a comprehensive alteration of host gut bacteriome in veterans with GWI, we then compared the association of differentially abundant species in the GWI cohort with fatigue symptoms as measured by the MFI fatigue total scores and the individual scales. MFI fatigue total score, fatigue average score, physical fatigue score, general fatigue score, mental fatigue score, reduced activity score, and reduced motivation score were all significantly increased in the veterans with GWI compared with controls (Figure 11A—table). A correlation analysis was conducted in GWI cases to determine the association of species abundance with fatigue total scores, fatigue average scores, physical fatigue score, and general fatigue scores. Results showed that *Lachnospiraceae* abundance and Chao 1 alpha diversity were negatively correlated with fatigue in the GWI cases (weak, R < 0.4) (*p* < 0.05) while the abundance of *Blautia* spp. was positively correlated with fatigue in the GWI cases (weak, R < +0.4) (*p* < 0.05).

## 4. Discussion

This is the first comprehensive report of microbiome dysbiosis in a large cohort of Gulf War veterans. The present study found that alpha diversity for GWI was lower and showed less inter-individual variability than controls but this was not significant. The beta diversity, which represents the difference in microbiome taxa between groups was significantly different in the GWI cases when compared to controls (*p* < 0.05). Further, the study found a statistically significant change in the diversity of species in veterans with GWI that were unique and presented a different niche of the microbiome when compared to the control group. Bioinformatic analysis using DESEQ2, MaAsLin, and Boruta further confirmed the uniqueness of the GWI veterans’ microbiome in identifying species signatures with an area under of the curve of 74.8% that can potentially be used as a predictor for GWI if further validated.

Our results did not find a significant difference in the alpha diversity of the two cohorts (GWI and control veterans, though a marked difference was observed); GWI cases had low alpha diversity but this was not statistically significant. Notably, alpha diversity shows richness and abundance (evenness) within a particular group, and a higher alpha diversity is often associated with good gut health. However, our result of a nonexistent difference may be due to a persistent pattern of the GWI microbiome, which was hardly perturbed by changes in diet or lifestyle or recent events for the 30 years that have passed since they participated in the war theater. It also may imply that GWI bacterial diversity is a unique signature that reflects a chronic condition. This notion is further confirmed from the results of the beta diversity analysis (Bray–Curtis analysis). Beta diversity, which shows the difference in abundance and richness between the GWI veterans and the control cohort, exhibited a significant difference at the family, genus, and species level (Figure 2 and Supplemental Figures S2 and S3) (*p* < 0.05). The significant change in beta diversity and unique clustering of the GWI microbiome reflected a unique signature, common to all GW deployed veterans studied in this cohort. Identifying this pattern opens up new therapeutic intervention strategies to restore the diversity similar to the control cohort. It may place more stress on dietary and endogenous gut metabolites as novel treatments. The above result of a significant difference in beta diversity between the GWI diseased and control clusters confirms that the GWI microbiome is a unique signature and reflects a change in the biome in symptomatic vs. non-symptomatic GW veterans.

Our results of a significant difference in the *Firmicutes*–*Bacteriodetes* ratio in GWI cases when compared to the control group confirm an association with a series of inflammatory triggers in other chronic conditions such as obesity and metabolic-associated steatotic liver disease (but not in MASH), which was reversed (high *Bacteroidetes*–*Firmicutes* ratio) in chronic fatigue syndrome [40,41,42]. Our results of a higher *Firmicutes*–*Bacteriodetes* ratio in the GWI cohort that also suffers from fatigue symptoms (in our study) and lower *Firmicutes*–*Bacteriodetes* in ME/CFS patients reported in prior studies may show the obvious difference in disease characteristics, symptom reporting, and etiology of GWI veterans compared to ME/CFS patients. Our results also showed a higher abundance of *Actinobacteria* phyla in the GWI cohort, often associated with gut homeostasis, but were not significantly altered, and the implications of such a result are unknown at this time. Interestingly, our previous study in a smaller cohort of GWI cases with GI disturbances showed a lower *Firmicutes* abundance, implying that GWI cases with a higher gastrointestinal inflammatory burden may reflect a unique disease subtype that needs to be studied in greater detail [10].

Due to the unclear characteristic pattern of examining the differences in the phylum, family, and genus abundance in the GWI disease phenotype, we used a series of bioinformatic tools to examine the species-level abundance and richness in this cohort. Our results of a differential abundance of *Blautia* sp., *Prevotella* sp., *Agathabacter* sp., *Bacteriodes uniformis*, and *Colinsella aerofa* reflected a unique species signature in the GWI cohort (Figure 3). A DESeq2 analysis more accurately identified a significant increase in the abundance of 63 species in GWI (Figure 4). A deeper linear analysis (Figure 5) showed a significant increase in bacterial species that are involved in a chronic inflammatory condition (*Blautia, streptococcus*, *Enterococcus faecium*, *Clostridium Perfringes*, *Klebsiella* sp., and *Escherichia Shigella Coli*). Notably, all of these species have been associated with a plethora of inflammatory diseases of diverse etiology, including bacteremia and irritable bowel syndrome (IBS) [43,44,45]. Our results also showed a significant decrease in short chain fatty acid (SCFA)-producing bacterial species contributing to a robust immune response and gut health (ref). Further, we used Boruta to identify a unique predictor of GWI via species identification (Figure 6 and Figure 7). A decrease in *Coprococcus* sp. and an increase in *Eisenbergiella* sp. were uniquely connected with GWI (Figure 6 and Figure 7). Notably, a decreased abundance of *Coprococcus* is associated with liver inflammation (NASH and cirrhosis) [45,46].

To validate whether these two species improve the identification of GWI, ROC curves were generated evaluating the ability of the entire microbiome to differentiate between GWI and control and that of only the unknown *Coprococcus* and *Eisenbergiella* species (Figure 8). At the taxonomic level, the entire community composition was not discriminative between the groups. However, reducing the community to only these two species improved the AUC to 74.8%. This may imply that these two taxa could serve as biomarkers of GWI if further validated.

*Coprococcus*, which shows significantly increased abundance in controls and decreased abundance in GWI, is a well-known commensal member of the gut community capable of producing beneficial SCFAs, specifically butyrate [47,48]. It is more frequently associated with positive roles in health, including decreased hyperactivity in children with ADHD, a reduction in constipation symptoms, and an improvement of rheumatoid arthritis symptoms [49,50]. Its increased abundance in controls means that its abundance is decreased in GWI in comparison, suggesting a loss of butyrate production capability. Treatment with butyrate has been shown to reduce inflammation in a GWI mouse model, suggesting that a reduction in species producing this SCFA may lead to gut inflammation [7].

*Eisenbergiella* is a less well-known genus but has been isolated from human stool [51]. A recent study in Japanese people found a positive association of *Eisenbergiella* with skeletal muscle mass, but another study found that it was associated with a risk of irritable bowel syndrome (IBS) [52,53]. It has also been found in increased abundance in patients with chronic pain, and, separately, patients with fibromyalgia, conditions which veterans with GWI often experience [54,55]. While future work is necessary to solidify a link between this genus/species and GWI, current evidence suggests that it could play an important role in disease pathology.

The functional capacity of the gut microbiome was predicted from this data using the tool PICRUSt [24]. Functional genes are not readily identified from 16S rRNA gene amplicon sequencing since this technique targets genes primarily for the identification of taxa present. Therefore, PICRUSt was developed in order to extrapolate the functional potential of microbial communities based on the taxa present. It does not directly identify the genes present, as is possible with whole genome sequencing. As such, some specificity in microbial functional capacity cannot be identified. However, functions were predicted using the Enzyme Commission (EC) enzyme and MetaCyc functional pathway databases (Figure 9) [39,56]. Due to the significant difference in beta diversity at the species level between GWI cases and controls, some functional differences were likely to be present. While there are no significant differences overall, community-level differences in alpha or beta diversity for either database, DESeq did identify 84 significantly enriched enzymes in GWI and 12 in controls. For MetaCyc pathways, only eight were enriched in GWI and none in the controls. An analysis of this data with MaAsLin2 did not identify any significantly enriched functions for either database. Still, Boruta identified three enzymes as important in differentiating between GWI and controls for EC enzymes only (Supplementary Figure S8). The three important enzymes are thiosulfate dehydrogenase, hydrogensulfite reductase, and L-xylulokinase.

As with the taxonomic data, an ROC curve was made to determine if all predicted enzymes improve the differentiation of the GWI and control groups. This resulted in an AUC of 57.9%, reflecting no ability of the data to differentiate between the groups. However, a reduction in the data to only the three important enzymes improved the AUC to 72.9%, suggesting that the abundance of these three enzymes may act as biomarkers in identifying GWI if further validated.

Both thiosulfate dehydrogenase (EC 1.8.2.2) and hydrogen sulfite reductase (EC 1.8.99.3) are oxidoreductase enzymes involved in the metabolism of hydrogen sulfide (H2S). Hydrogen sulfite reductase has since been renamed to dissimilatory sulfite reductase (EC 1.8.1.22). Thiosulfate dehydrogenase can recycle thiosulfate to H2S while dissimilatory sulfite reductase catalyzes the last step of sulfate and taurine respiration to form H2S [57,58]. While the production of H_2_S by the gut microbiota is normal, its concentration and source determine whether it has beneficial or toxic health effects [59]. At low endogenous concentrations, it has been found to have antihypertensive properties; it stabilizes the mucus layers of the gut epithelium, prevents adherence of biofilms to the epithelium, inhibits the release of pathobionts, and helps resolve inflammation and injury to the tissue [59]. At high concentrations, H_2_S can disrupt the mucus layer, induce inflammation, and contribute to the development of colorectal cancer [58,59]. Both enzymes are predicted to be present at higher abundance in the controls than in GWI subjects. If GWI subjects lack sufficient production of these enzymes, they may not be able to adequately produce H_2_S, leading to a loss of this metabolite’s beneficial properties. This may be a contributor to the gut dysfunction reported in a subset of veterans with GWI.

L-xylulokinase (EC 2.7.1.53) is an enzyme involved in xylose metabolism. Xylose is the second most abundant sugar in nature and the first step in its metabolism by microorganisms is conversion to xylulose by D- and L-xylulose kinases [60]. An increased abundance of this kinase may suggest increased xylose metabolism. Xylose has been shown to induce the production of prophages in E. coli, which could be used as a targeted antimicrobial therapy [61]. L-xylulokinase was higher in the GWI group than controls, implying a higher metabolic rate of xylose in these veterans. Previous research has shown that the sweetener xylitol can be metabolized to xylose through the pathway involving L-xylulokinase, producing SCFAs [62]. This decreases the pH in the gut, reducing potential pathogenic species such as Escherichia and Staphylococcus. An increase in the abundance of L-xylulokinase may suggest that veterans with GWI have the potential to produce more beneficial SCFAs if given the proper substrates, which may help improve symptoms. A deep analysis of metabolic pathways by DESEQ2 showed a significant enrichment of the methylaspartate cycle and glycolysis in GWI cases but the significance of such a change remains unknown at this time (Figure 10).

While differences in the gut microbiome between veterans with GWI and controls are subtle, there are significant changes that could be targeted to aid in the treatment of GWI and increase understanding of disease pathology. An overall community composition shift towards similarity among the microbiota of GWI veterans suggests a common mechanism driving microbiome changes in the disease. Machine learning methods suggest that *Eisenbergiella* and *Coprococcus* are key players in these changes and may be significant disease markers. Enzymes involved in hydrogen sulfide and SCFA metabolism hint at possible mechanisms of action involved in the disease. *Coprococcus* is a known SCFA producer, although it was found to be higher in controls, while L-xylulokinase was found to be higher in GWI. While *Eisenbergiella* has been associated with specific conditions, literature on its metabolic pathways is sparse.

GWI is consistently associated with chronic fatigue symptoms and has remained an understudied area in the field [1,2]. Further, bioenergetic impairment has been recorded in veterans with GWI and has ties to chronic fatigue symptoms [63,64]. Through our comprehensive analysis of the gut microbiome, we aimed to associate the changes in bacterial diversity with fatigue scores. Our results of a correlation, albeit a weak one (Figure 11A–E) between fatigue scores and host bacterial abundance indicate that gut microbiome dysbiosis is associated with the consistent fatigue reported in clinical studies [65]. Our findings of a correlation between fatigue scores and gut bacteria (low abundance of *Lachnospiraceae*) align with another study that found that mental energy, mental fatigue, physical energy, and physical fatigue were significantly associated with levels of *Lachnospiraceae* [66]. Our correlation indices were weak (R < 0.4). However, they were significant (*p* < 0.05), and a higher sample number in future studies may help elucidate the above connection with chronic fatigue in veterans with GWI.

### Limitations

Although this study with a large cohort, the first-ever comprehensive study involving GWI veterans and gut dysbiosis, has shown promising links between specific microbes, their functions, and GWI and a connection with chronic fatigue, it is limited by the size of the cohort and sequencing technique used. The stool samples were collected at home by the study participants with storage temperature and transport methods not controlled, which could have introduced some variance in the samples. Fatigue in GWI is a symptom associated with multi-organ etiology, such as gastrointestinal disturbances, cognitive difficulties, mitochondrial bioenergetics, and post-exertional malaise. The present study did not factor in the above causes and may need to be investigated separately in sub cohorts to better understand the GWI multisystem pathology. The host gut microbiome may connect with each of the pathologies noted above and may help design treatment strategies. The host gut microbiome is also affected by diet and long-term medications. A future study should also consider these existing lifestyle alterations in microbiome dysbiosis and could compare non-deployed and other illness groups. 16S rRNA amplicon sequencing is a very affordable option to profile microbial communities, but it is mostly limited to identifying microbes. In this study, the tool PICRUSt was used to estimate the functional potential of the GWI community, providing hints to important functional differences that could provide mechanistic insight into GWI and its treatment. Future work would benefit from the use of whole genome sequencing, where the entire genetic content of the gut microbiome in veterans with GWI could be sequenced, providing a vast amount of information on bacteria down to the sub-strain level as well as the specific identification of the presence of functional genes and pathways and even profiling of other kingdoms (fungi, protists, phages, etc.). This more in-depth method of sequencing would provide a much fuller picture of the gut microbiome and its functionality in veterans with GWI, providing a wholistic, systems-level understanding of the complexities of the microbiome in GWI and how it might be leveraged to improve the quality of life for veterans.

## 5. Conclusions

This study is the most comprehensive to date that establishes an association of gut microbiome dysbiosis with Gulf War veterans who were deployed in the war theater and specific biomarkers that may be predictive of GWI case status. The study also suggests an association between dysbiosis and chronic fatigue, though more in-depth studies are needed to evaluate further the wider nature of fatigue experienced in GW veterans. Fatigue is associated with multiple facets of human pathology, and areas such as immunosenescence, aging, gastrointestinal disturbances, and memory deficits need to be studied in greater detail if a deeper understanding of its mechanisms is to be pursued. The results also suggest potential therapeutic targets for veterans with GWI that target the gut microbiome and specific symptoms of the illness.

## Figures and Tables

**Table 1:** Demographics.

		Overall	Case or Control Status	
			Control	GWI (Cases)
**Total (N)**		89	26	63
**1. Age** **2. Sex**	**Mean (Standard Deviation)** **1: Male** **2: Female**	57 (7) 71 (79.8%) 18 (20.2%)	60 (10) 24 (92.3%) 2 (7.7%)	56 (6) 47 (74.6%) 16 (25.4%)
**3. Hispanic or Latino descent**	**1: Yes**	11 (12.8%)	1 (4.0%)	10 (16.4%)
	**2: No**	75 (87.2%)	24 (96.0%)	51 (83.6%)
**4. Race**	**1: Black/African American**	9 (10.1%)	2 (7.7%)	7 (11.1%)
	**2: White/Caucasian**	70 (78.7%)	23 (88.5%)	47 (74.6%)
	**3: Asian/Pacific Islander**	2 (2.2%)	0 (0.0%)	2 (3.2%)
	**4: Aleutian, Eskimo or American Indian**	2 (2.2%)	1 (3.8%)	1 (1.6%)
	**5: Other Multiracial**	6 (6.7%)	0 (0.0%)	6 (9.5%)
**5. Total years of education**		15 (2)	16 (2)	15 (2)

Note: No significant differences were found between cases and controls.

## Data Availability

The data presented in this study are available on request from the corresponding author.

## Supplementary Materials

The following supporting information can be downloaded at: https://www.mdpi.com/article/10.3390/ijerph21081102/s1, Figure S1: α-diversity analysis (Shannon Index) of altered gut bacteriome in the control group (deployed GW Veterans with no GWI) and the GWI group (deployed GW veterans with GWI symptoms). *p* < 0.05 was considered as statistically significant; Figure S2: β-diversity analysis (Bray–Curtis) of altered gut bacteriome at Family- Bacteria level in the control group (deployed GW Veterans with no GWI) and the GWI group (deployed GW veterans with GWI symptoms). *p* < 0.05 was considered as statistically significant; Figure S3: β-diversity analysis (Bray–Curtis) of altered gut bacteriome at Genus- Bacteria level in the control group (deployed GW Veterans with no GWI) and the GWI group (deployed GW veterans with GWI symptoms). *p* < 0.05 was considered as statistically significant; Figure S4: Stacked bar representation of altered gut bacteriome at phylum level in the control group (deployed GW veterans with no GWI) and the GWI group (deployed GW veterans with GWI symptoms) [at Cohort level]; Figure S5: Stacked bar representation of altered relative abundance of bacteriome at Genus level in the control group (deployed GW veterans with no GWI) and the GWI group (deployed GW veterans with GWI symptoms). *p* < 0.05 was considered as statistically significant; Figure S6: DESeq2 plot shows enriched taxa with log2 fold change plotted against log2 abundance at Genus level in control groups (deployed GW veterans with no GWI) and GWI group (deployed GW veterans with GWI symptoms). The red points show features significantly enriched in the GWI group and blue points show features significantly enriched in the control group. *p* < 0.05 was considered as statistically significant; Figure S7: Stacked bar representation of altered Enzyme Commission terms in the control group (deployed GW veterans with no GWI) and the GWI group (deployed GW veterans with GWI symptoms); Figure S8: Table and plot showing all Enzyme Commission enzymes ranked by order of importance according to the Boruta algorithm. Of all enzymes identified, three were important in identifying GWI samples (labeled in green in the plot). The features in yellow are tentative and those in red are rejected as important.

## References

[1] Mawson A.R., Croft A.M. (2019). Gulf War Illness: Unifying Hypothesis for a Continuing Health Problem. Int. J. Environ. Res. Public Health.

[2] White R.F., Steele L., O’Callaghan J.P., Sullivan K., Binns J.H., Golomb B.A., Bloom F.E., Bunker J.A., Crawford F., Graves J.C. (2016). Recent research on Gulf War illness and other health problems in veterans of the 1991 Gulf War: Effects of toxicant exposures during deployment. Cortex.

[3] Cohen J., Mathew A., Dourvetakis K.D., Sanchez-Guerrero E., Pangeni R.P., Gurusamy N., Aenlle K.K., Ravindran G., Twahir A., Isler D. (2024). Recent Research Trends in Neuroinflammatory and Neurodegenerative Disorders. Cells.

[4] Golomb B.A. (2008). Acetylcholinesterase inhibitors and Gulf War illnesses. Proc. Natl. Acad. Sci. USA.

[5] Alhasson F., Das S., Seth R., Dattaroy D., Chandrashekaran V., Ryan C.N., Chan L.S., Testerman T., Burch J., Hofseth L.J. (2017). Altered gut microbiome in a mouse model of Gulf War Illness causes neuroinflammation and intestinal injury via leaky gut and TLR4 activation. PLoS ONE.

[6] Chatterjee S., Bose D., Seth R. (2021). Host gut microbiome and potential therapeutics in Gulf War Illness: A short review. Life Sci..

[7] Seth R.K., Kimono D., Alhasson F., Sarkar S., Albadrani M., Lasley S.K., Horner R., Janulewicz P., Nagarkatti M., Nagarkatti P. (2018). Increased butyrate priming in the gut stalls microbiome associated-gastrointestinal inflammation and hepatic metabolic reprogramming in a mouse model of Gulf War Illness. Toxicol. Appl. Pharmacol..

[8] Kimono D., Sarkar S., Albadrani M., Seth R., Bose D., Mondal A., Li Y., Kar A.N., Nagarkatti M., Nagarkatti P. (2019). Dysbiosis-Associated Enteric Glial Cell Immune-Activation and Redox Imbalance Modulate Tight Junction Protein Expression in Gulf War Illness Pathology. Front. Physiol..

[9] Seth R.K., Maqsood R., Mondal A., Bose D., Kimono D., Holland L.A., Lloyd P.J., Klimas N., Horner R.D., Sullivan K. (2019). Gut DNA Virome Diversity and Its Association with Host Bacteria Regulate Inflammatory Phenotype and Neuronal Immunotoxicity in Experimental Gulf War Illness. Viruses.

[10] Janulewicz P.A., Seth R.K., Carlson J.M., Ajama J., Quinn E., Heeren T., Klimas N., Lasley S.M., Horner R.D., Sullivan K. (2019). The Gut-Microbiome in Gulf War Veterans: A Preliminary Report. Int. J. Environ. Res. Public Health.

[11] Kimono D., Bose D., Seth R.K., Mondal A., Saha P., Janulewicz P., Sullivan K., Lasley S., Horner R., Klimas N. (2020). Host Akkermansia muciniphila Abundance Correlates with Gulf War Illness Symptom Persistence via NLRP3-Mediated Neuroinflammation and Decreased Brain-Derived Neurotrophic Factor. Neurosci. Insights.

[12] Bose D., Mondal A., Saha P., Kimono D., Sarkar S., Seth R.K., Janulewicz P., Sullivan K., Horner R., Klimas N. (2020). TLR Antagonism by Sparstolonin B Alters Microbial Signature and Modulates Gastrointestinal and Neuronal Inflammation in Gulf War Illness Preclinical Model. Brain Sci..

[13] Dinan T.G., Cryan J.F. (2017). The Microbiome-Gut-Brain Axis in Health and Disease. Gastroenterol. Clin. N. Am..

[14] Mayer E.A., Tillisch K., Gupta A. (2015). Gut/brain axis and the microbiota. J. Clin. Investig..

[15] Malhotra D., Boyle S.H., Gifford E.J., Sullivan B.A., Wenker T.H.N., Abs N., Ahmed S.T., Upchurch J., Vahey J., Stafford C. (2023). Self-reported gastrointestinal disorders among veterans with gulf war illness with and without posttraumatic stress disorder. Neurogastroenterol. Motil..

[16] Collier C.A., Salikhova A., Sabir S., Foncerrada S., Raghavan S.A. (2024). Crisis in the gut: Navigating gastrointestinal challenges in Gulf War Illness with bioengineering. Mil. Med. Res..

[17] Mukhopadhya I., Segal J.P., Carding S.R., Hart A.L., Hold G.L. (2019). The gut virome: The ‘missing link’ between gut bacteria and host immunity?. Ther. Adv. Gastroenterol..

[18] Keating D., Zundel C., Abreu M., Krengel M., Aenlle K., Nichols M.D., Toomey R., Chao L., Golier J., Abdullah L. (2021). Boston biorepository, recruitment and integrative network (BBRAIN): A resource for the Gulf War Illness scientific community. Life Sci..

[19] Steele L. (2000). Prevalence and patterns of Gulf War illness in Kansas veterans: Association of symptoms with characteristics of person, place, and time of military service. Am. J. Epidemiol..

[20] Sultana E., Shastry N., Kasarla R., Hardy J., Collado F., Aenlle K., Abreu M., Sisson E., Sullivan K., Klimas N. (2024). Disentangling the effects of PTSD from Gulf War Illness in male veterans via a systems-wide analysis of immune cell, cytokine, and symptom measures. Mil. Med. Res..

[21] Callahan B.J., Mcmurdie P.J., Rosen M.J., Han A.W., Johnson A.J.A., Holmes S.P. (2016). DADA2: High-resolution sample inference from Illumina amplicon data. Nat. Methods.

[22] Straub D., Blackwell N., Langarica-Fuentes A., Peltzer A., Nahnsen S., Kleindienst S. (2020). Interpretations of Environmental Microbial Community Studies Are Biased by the Selected 16S rRNA (Gene) Amplicon Sequencing Pipeline. Front. Microbiol..

[23] Li M., Kopylova E., Mao J., Namkoong J., Sanders J., Wu J. (2024). Microbiome and lipidomic analysis reveal the interplay between skin bacteria and lipids in a cohort study. Front. Microbiol..

[24] Douglas G.M., Maffei V.J., Zaneveld J.R., Yurgel S.N., Brown J.R., Taylor C.M., Huttenhower C., Langille M.G.I. (2020). PICRUSt2 for prediction of metagenome functions. Nat. Biotechnol..

[25] Karp P.D., Riley M., Paley S.M., Pellegrini-Toole A. (2002). The MetaCyc Database. Nucleic Acids Res..

[26] Zhao H., Sun R., Wu L., Huang P., Liu W., Ma Q., Liao Q., Du J. (2024). Bioinformatics Identification and Experimental Validation of a Prognostic Model for the Survival of Lung Squamous Cell Carcinoma Patients. Biochem. Genet..

[27] Paradis E., Schliep K. (2019). Ape 5.0: An environment for modern phylogenetics and evolutionary analyses in R. Bioinformatics.

[28] Love M.I., Huber W., Anders S. (2014). Moderated estimation of fold change and dispersion for RNA-seq data with DESeq2. Genome Biol..

[29] Mallick H., Rahnavard A., McIver L.J., Ma S., Zhang Y., Nguyen L.H., Tickle T.L., Weingart G., Ren B., Schwager E.H. (2021). Multivariable association discovery in population-scale meta-omics studies. PLoS Comput. Biol..

[30] Robin X., Turck N., Hainard A., Tiberti N., Lisacek F., Sanchez J.-C., Müller M. (2011). pROC: An open-source package for R and S+ to analyze and compare ROC curves. BMC Bioinform..

[31] Willis A.D. (2019). Rarefaction, Alpha Diversity, and Statistics. Front. Microbiol..

[32] Su X. (2021). Elucidating the Beta-Diversity of the Microbiome: From Global Alignment to Local Alignment. mSystems.

[33] Santos-Marcos J.A., Perez-Jimenez F., Camargo A. (2019). The role of diet and intestinal microbiota in the development of metabolic syndrome. J. Nutr. Biochem..

[34] Duan R., Zhu S., Wang B., Duan L. (2019). Alterations of Gut Microbiota in Patients with Irritable Bowel Syndrome Based on 16S rRNA-Targeted Sequencing: A Systematic Review. Clin. Transl. Gastroenterol..

[35] Hsu Y.-J., Huang W.-C., Lin J.-S., Chen Y.-M., Ho S.-T., Huang C.-C., Tung Y.-T. (2018). Kefir Supplementation Modifies Gut Microbiota Composition, Reduces Physical Fatigue, and Improves Exercise Performance in Mice. Nutrients.

[36] Degenhardt F., Seifert S., Szymczak S. (2019). Evaluation of variable selection methods for random forests and omics data sets. Brief Bioinform..

[37] Guo P., Luo Y., Mai G., Zhang M., Wang G., Zhao M., Gao L., Li F., Zhou F. (2014). Gene expression profile based classification models of psoriasis. Genomics.

[38] Saulnier D.M., Riehle K., Mistretta T.A., Diaz M.A., Mandal D., Raza S., Weidler E.M., Qin X., Coarfa C., Milosavljevic A. (2011). Gastrointestinal microbiome signatures of pediatric patients with irritable bowel syndrome. Gastroenterology.

[39] Caspi R., Billington R., Keseler I.M., Kothari A., Krummenacker M., Midford P.E., Ong W.K., Paley S., Subhraveti P., Karp P.D. (2020). The MetaCyc database of metabolic pathways and enzymes—A 2019 update. Nucleic Acids Res..

[40] Zhu L., Baker S.S., Gill C., Liu W., Alkhouri R., Baker R.D., Gill S.R. (2013). Characterization of gut microbiomes in nonalcoholic steatohepatitis (NASH) patients: A connection between endogenous alcohol and NASH. Hepatology.

[41] Ley R.E., Turnbaugh P.J., Klein S., Gordon J.I. (2006). Microbial ecology: Human gut microbes associated with obesity. Nature.

[42] Hoozemans J., de Brauw M., Nieuwdorp M., Gerdes V. (2021). Gut Microbiome and Metabolites in Patients with NAFLD and after Bariatric Surgery: A Comprehensive Review. Metabolites.

[43] Shimasaki T., Seekatz A., Bassis C., Rhee Y., Yelin R.D., Fogg L., Dangana T., Cisneros E.C., A Weinstein R., Okamoto K. (2019). Increased Relative Abundance of *Klebsiella pneumoniae* Carbapenemase-producing *Klebsiella pneumoniae* within the Gut Microbiota Is Associated with Risk of Bloodstream Infection in Long-term Acute Care Hospital Patients. Clin. Infect. Dis..

[44] Shaikh S.D., Sun N., Canakis A., Park W.Y., Weber H.C. (2023). Irritable Bowel Syndrome and the Gut Microbiome: A Comprehensive Review. J. Clin. Med..

[45] Piazzesi A., Putignani L. (2022). Extremely small and incredibly close: Gut microbes as modulators of inflammation and targets for therapeutic intervention. Front. Microbiol..

[46] Wang T., Guo X.K., Xu H. (2021). Disentangling the Progression of Non-alcoholic Fatty Liver Disease in the Human Gut Microbiota. Front. Microbiol..

[47] Louis P., Flint H.J. (2017). Formation of propionate and butyrate by the human colonic microbiota. Environ. Microbiol..

[48] Ohira H., Tsutsui W., Fujioka Y. (2017). Are Short Chain Fatty Acids in Gut Microbiota Defensive Players for Inflammation and Atherosclerosis?. J. Atheroscler. Thromb..

[49] Pan R., Wang L., Xu X., Chen Y., Wang H., Wang G., Zhao J., Chen W. (2022). Crosstalk between the Gut Microbiome and Colonic Motility in Chronic Constipation: Potential Mechanisms and Microbiota Modulation. Nutrients.

[50] Szopinska-Tokov J., Dam S., Naaijen J., Konstanti P., Rommelse N., Belzer C., Buitelaar J., Franke B., Bloemendaal M., Aarts E. (2020). Investigating the Gut Microbiota Composition of Individuals with Attention-Deficit/Hyperactivity Disorder and Association with Symptoms. Microorganisms.

[51] Togo A., Khelaifia S., Bittar F., Maraninchi M., Raoult D., Million M. (2016). ‘*Eisenbergiella massiliensis*’, a new species isolated from human stool collected after bariatric surgery. New Microbes New Infect..

[52] Liu B., Ye D., Yang H., Song J., Sun X., He Z., Mao Y., Hao G. (2023). Assessing the relationship between gut microbiota and irritable bowel syndrome: A two-sample Mendelian randomization analysis. BMC Gastroenterol..

[53] Sugimura Y., Kanda A., Sawada K., Wai K.M., Tanabu A., Ozato N., Midorikawa T., Hisada T., Nakaji S., Ihara K. (2022). Association between Gut Microbiota and Body Composition in Japanese General Population: A Focus on Gut Microbiota and Skeletal Muscle. Int. J. Environ. Res. Public Health.

[54] Goudman L., Demuyser T., Pilitsis J.G., Billot M., Roulaud M., Rigoard P., Moens M. (2024). Gut dysbiosis in patients with chronic pain: A systematic review and meta-analysis. Front. Immunol..

[55] Minerbi A., Fitzcharles M.A. (2020). Gut microbiome: Pertinence in fibromyalgia. Clin. Exp. Rheumatol..

[56] Caspi R., Billington R., Ferrer L., Foerster H., Fulcher C.A., Keseler I.M., Kothari A., Krummenacker M., Latendresse M., Mueller L.A. (2016). The MetaCyc database of metabolic pathways and enzymes and the BioCyc collection of pathway/genome databases. Nucleic Acids Res..

[57] Tomasova L., Grman M., Ondrias K., Ufnal M. (2021). The impact of gut microbiota metabolites on cellular bioenergetics and cardiometabolic health. Nutr. Metab..

[58] Wolf P.G., Cowley E.S., Breister A., Matatov S., Lucio L., Polak P., Ridlon J.M., Gaskins H.R., Anantharaman K. (2022). Diversity and distribution of sulfur metabolic genes in the human gut microbiome and their association with colorectal cancer. Microbiome.

[59] Buret A.G., Allain T., Motta J.P., Wallace J.L. (2022). Effects of Hydrogen Sulfide on the Microbiome: From Toxicity to Therapy. Antioxid. Redox Signal..

[60] Zhao Z., Xian M., Liu M., Zhao G. (2020). Biochemical routes for uptake and conversion of xylose by microorganisms. Biotechnol. Biofuels.

[61] Hu J., Wu Y., Kang L., Liu Y., Ye H., Wang R., Zhao J., Zhang G., Li X., Wang J. (2023). Dietary D-xylose promotes intestinal health by inducing phage production in *Escherichia coli*. NPJ Biofilms Microbiomes.

[62] Xiang S., Ye K., Li M., Ying J., Wang H., Han J., Shi L., Xiao J., Shen Y., Feng X. (2021). Xylitol enhances synthesis of propionate in the colon via cross-feeding of gut microbiota. Microbiome.

[63] Golomb B.A., Han J.H., Fung A., Berg B.K., Miller B.J., Hamilton G. (2024). Bioenergetic impairment in Gulf War illness assessed via (31)P-MRS. Sci. Rep..

[64] Sundberg C.W., Fitts R.H. (2019). Bioenergetic basis of skeletal muscle fatigue. Curr. Opin. Physiol..

[65] Wylie G., Genova H., Dobryakova E., DeLuca J., Chiaravalloti N., Falvo M., Cook D. (2019). Fatigue in Gulf War Illness is associated with tonically high activation in the executive control network. Neuroimage Clin..

[66] Boolani A., Gallivan K.M., Ondrak K.S., Christopher C.J., Castro H.F., Campagna S.R., Taylor C.M., Luo M., Dowd S.E., Smith M.L. (2022). Trait Energy and Fatigue May Be Connected to Gut Bacteria among Young Physically Active Adults: An Exploratory Study. Nutrients.

