# Supplementary material for: Gulf War Illness Is Associated with Host Gut Microbiome Dysbiosis and Is Linked to Altered Species Abundance in Veterans from the BBRAIN Cohort
Source: Int J Environ Res Public Health. 2024 Aug 21;21(8):1102. doi: 10.3390/ijerph21081102 (PMC11354743; doi:10.3390/ijerph21081102)
Supplement: Supplementary file 1 [file ijerph-21-01102-s001.zip › ijerph-3076198-supplementary.pptx]

## Slide 1
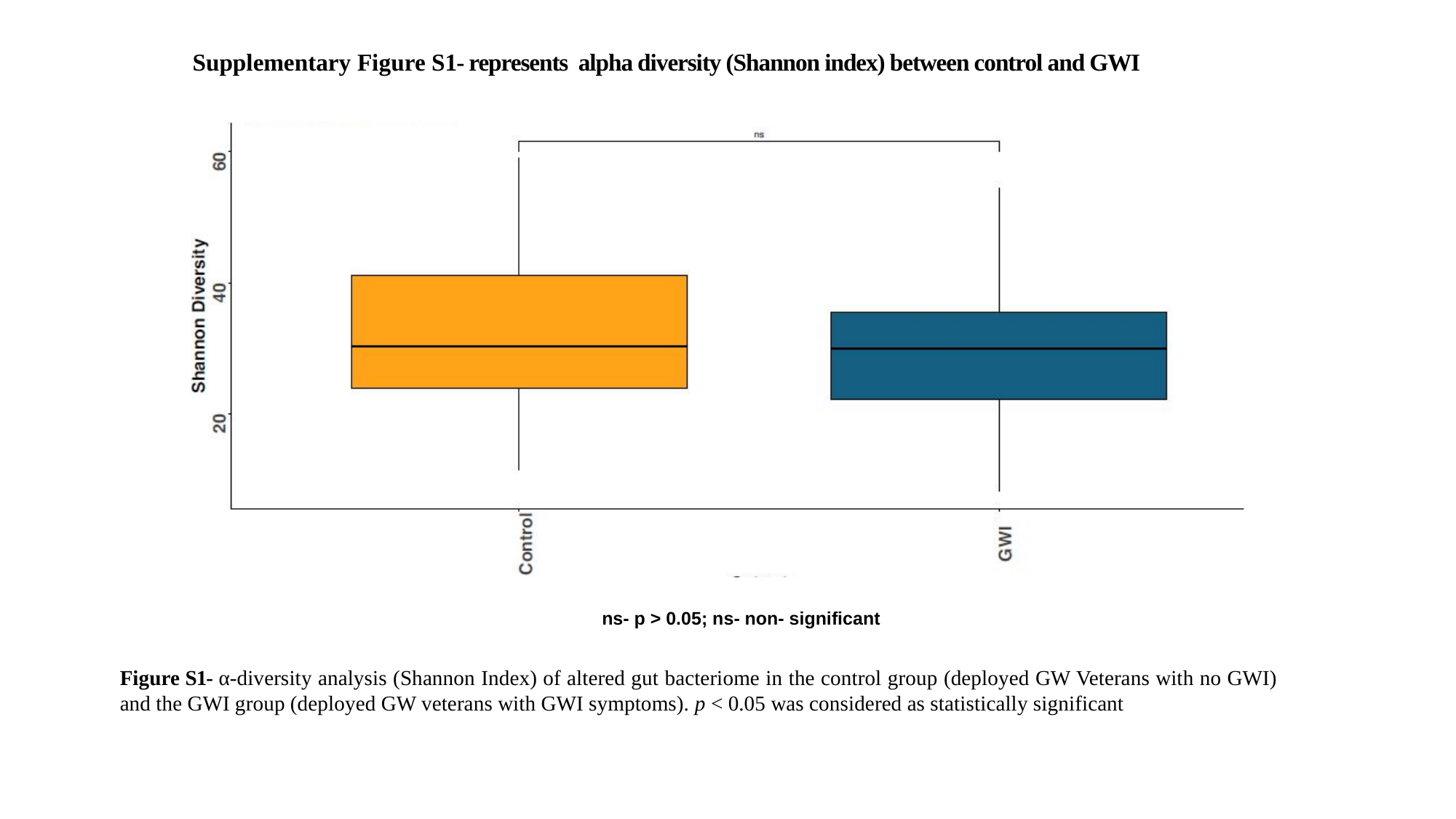

Supplementary Figure S1- represents alpha diversity (Shannon index) between control and GWI
ns- p > 0.05; ns- non- significant
Figure S1- α-diversity analysis (Shannon Index) of altered gut bacteriome in the control group (deployed GW Veterans with no GWI) and the GWI group (deployed GW veterans with GWI symptoms). p < 0.05 was considered as statistically significant

## Slide 2
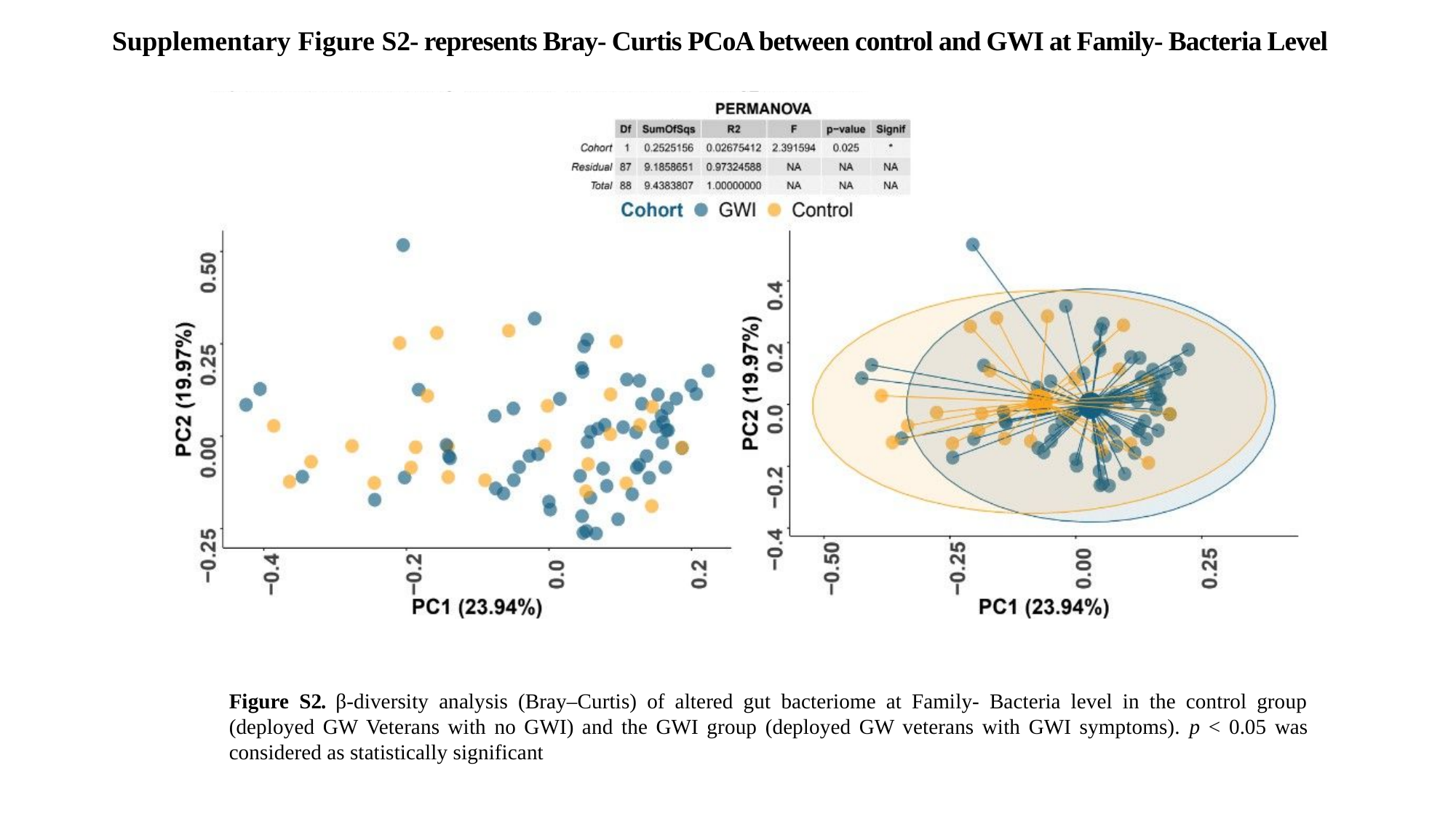

Supplementary Figure S2- represents Bray- Curtis PCoA between control and GWI at Family- Bacteria Level
Figure S2. β-diversity analysis (Bray–Curtis) of altered gut bacteriome at Family- Bacteria level in the control group (deployed GW Veterans with no GWI) and the GWI group (deployed GW veterans with GWI symptoms). p < 0.05 was considered as statistically significant

## Slide 3
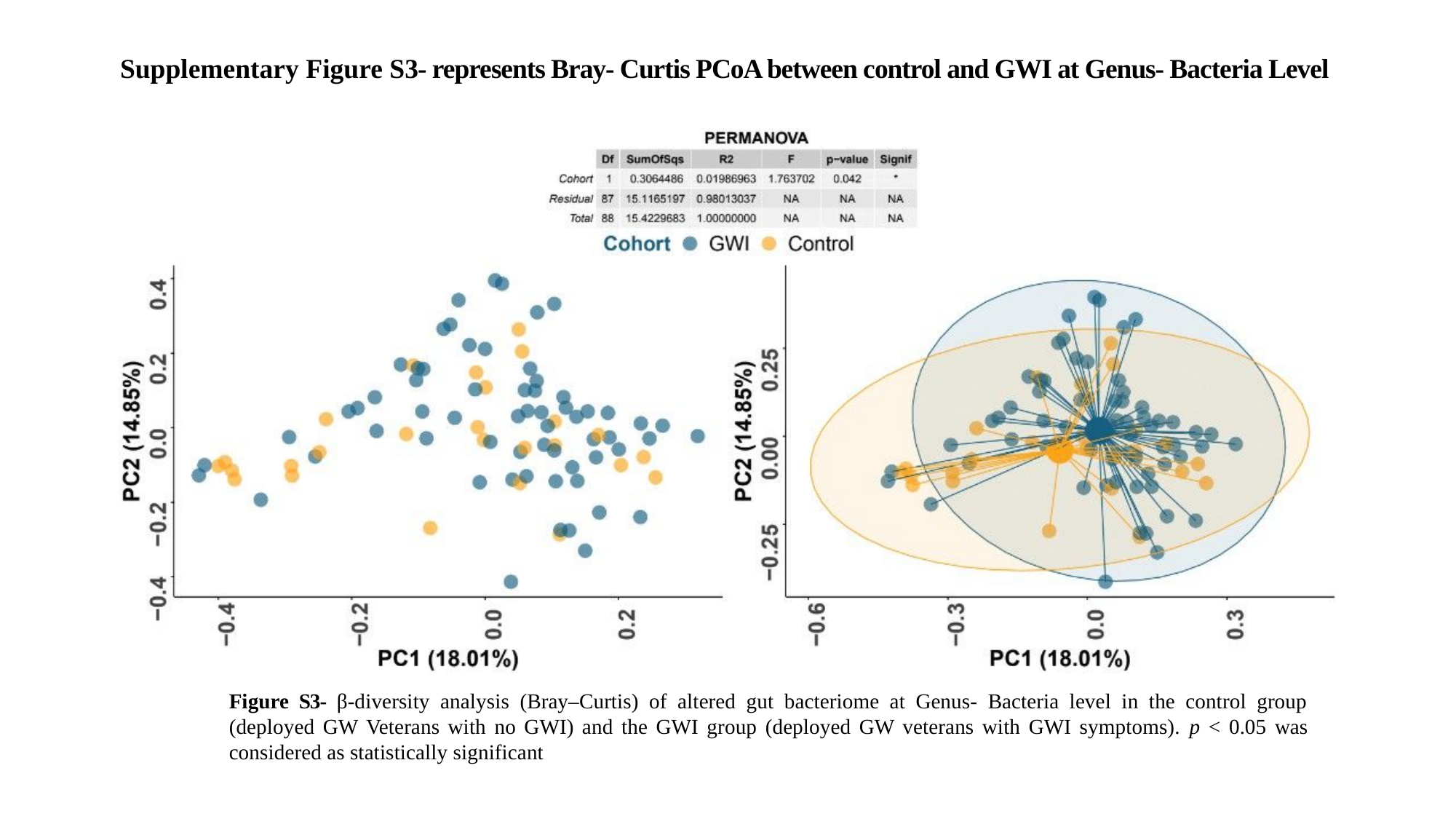

Supplementary Figure S3- represents Bray- Curtis PCoA between control and GWI at Genus- Bacteria Level
Figure S3- β-diversity analysis (Bray–Curtis) of altered gut bacteriome at Genus- Bacteria level in the control group (deployed GW Veterans with no GWI) and the GWI group (deployed GW veterans with GWI symptoms). p < 0.05 was considered as statistically significant

## Slide 4
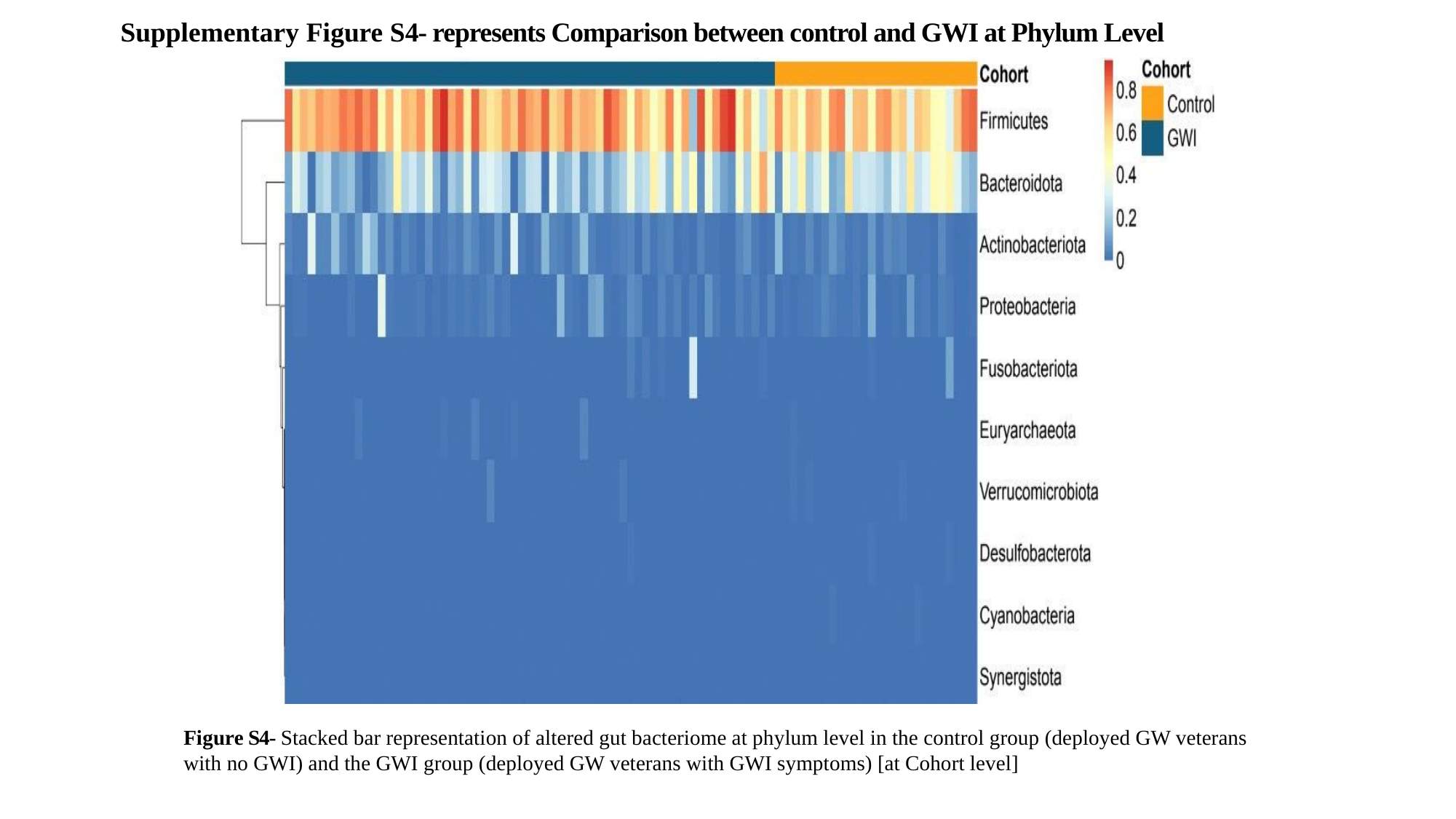

Supplementary Figure S4- represents Comparison between control and GWI at Phylum Level
Figure S4- Stacked bar representation of altered gut bacteriome at phylum level in the control group (deployed GW veterans with no GWI) and the GWI group (deployed GW veterans with GWI symptoms) [at Cohort level]

## Slide 5
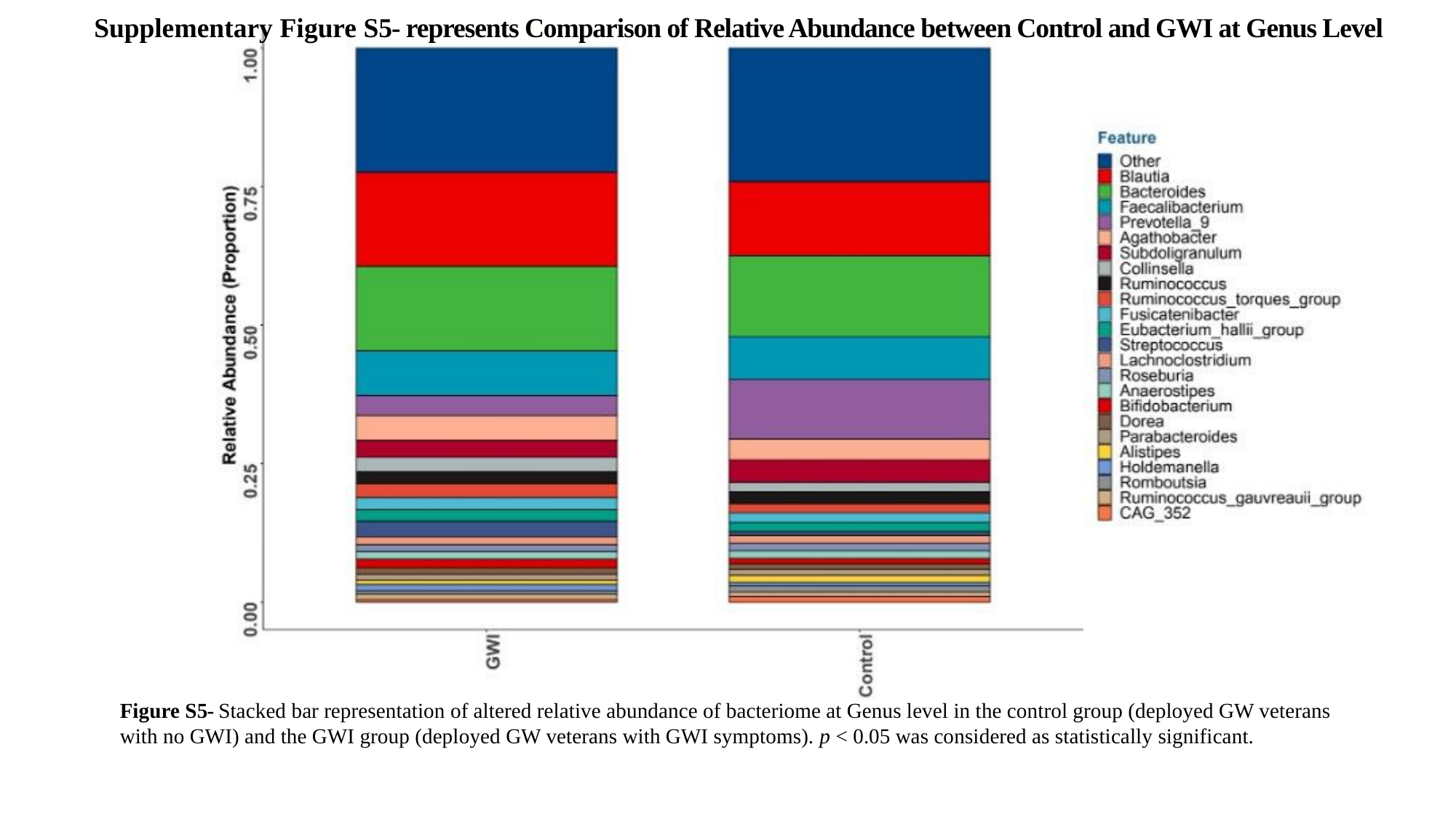

Supplementary Figure S5- represents Comparison of Relative Abundance between Control and GWI at Genus Level
Figure S5- Stacked bar representation of altered relative abundance of bacteriome at Genus level in the control group (deployed GW veterans with no GWI) and the GWI group (deployed GW veterans with GWI symptoms). p < 0.05 was considered as statistically significant.

## Slide 6
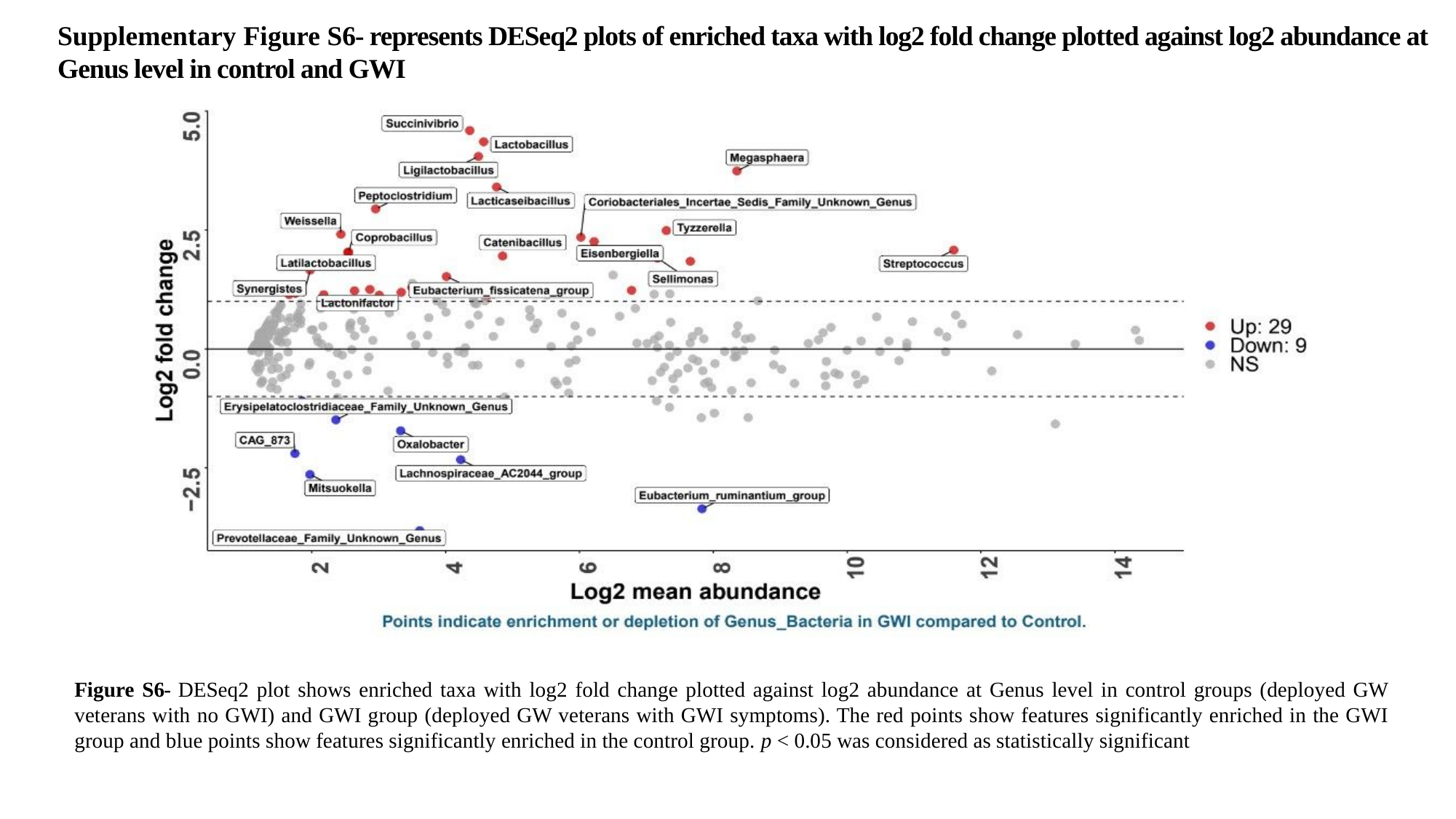

Supplementary Figure S6- represents DESeq2 plots of enriched taxa with log2 fold change plotted against log2 abundance at Genus level in control and GWI
Figure S6- DESeq2 plot shows enriched taxa with log2 fold change plotted against log2 abundance at Genus level in control groups (deployed GW veterans with no GWI) and GWI group (deployed GW veterans with GWI symptoms). The red points show features significantly enriched in the GWI group and blue points show features significantly enriched in the control group. p < 0.05 was considered as statistically significant

## Slide 7
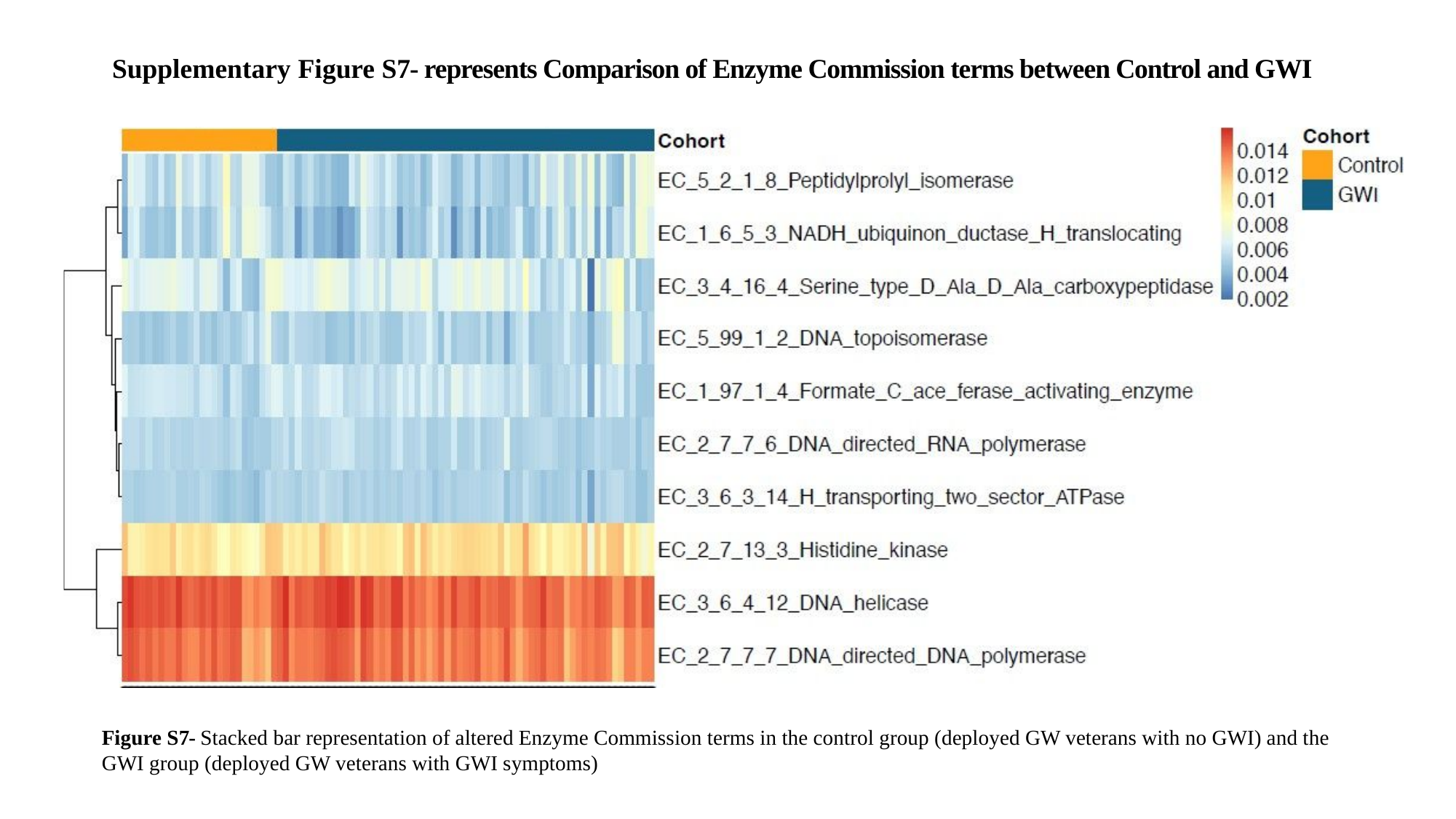

Supplementary Figure S7- represents Comparison of Enzyme Commission terms between Control and GWI
Figure S7- Stacked bar representation of altered Enzyme Commission terms in the control group (deployed GW veterans with no GWI) and the GWI group (deployed GW veterans with GWI symptoms)

## Slide 8
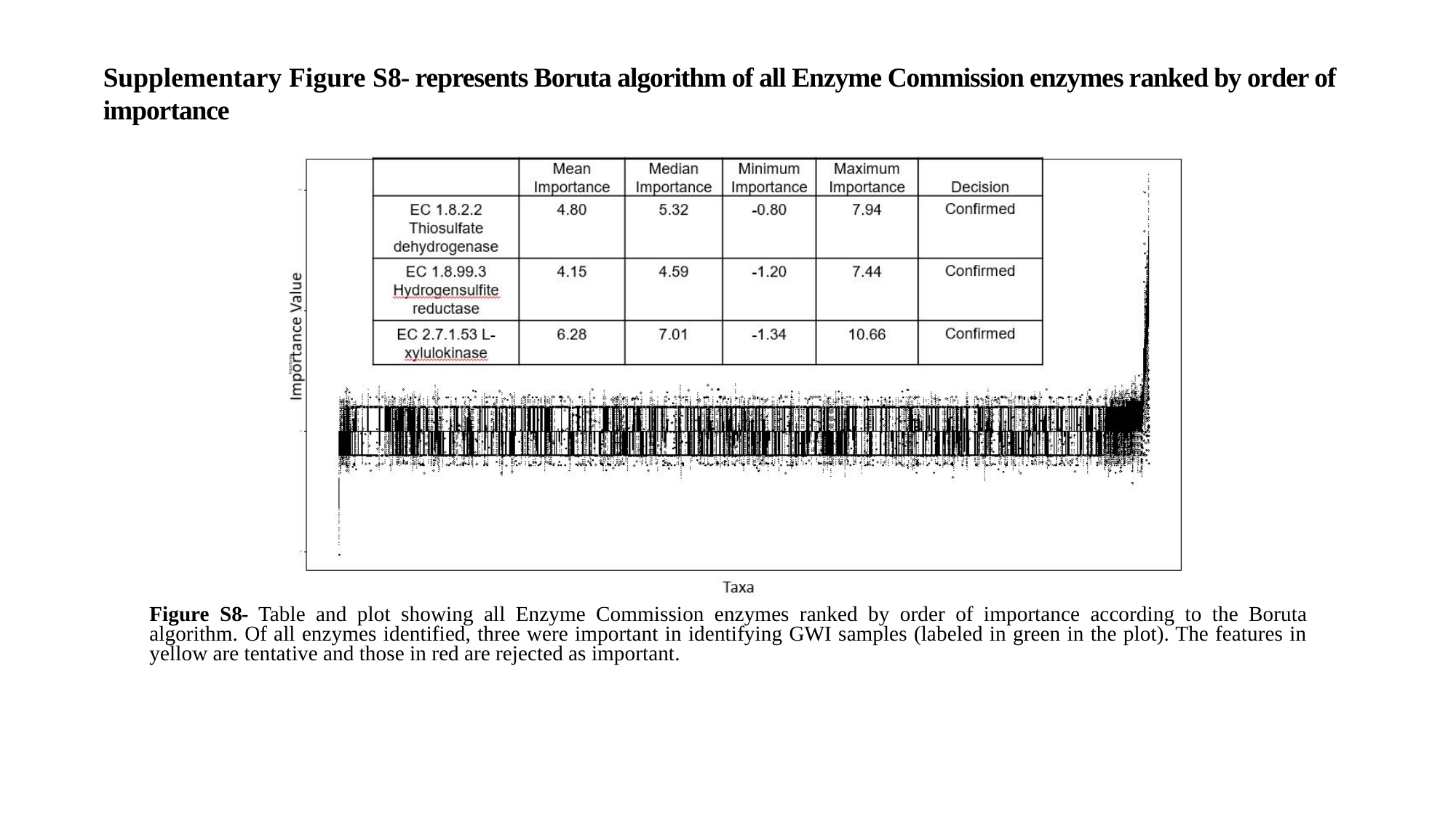

Supplementary Figure S8- represents Boruta algorithm of all Enzyme Commission enzymes ranked by order of importance
Figure S8- Table and plot showing all Enzyme Commission enzymes ranked by order of importance according to the Boruta algorithm. Of all enzymes identified, three were important in identifying GWI samples (labeled in green in the plot). The features in yellow are tentative and those in red are rejected as important.
